# Risk of stroke under disease modifying therapies for multiple sclerosis: a systematic review

**DOI:** 10.1177/17562864251321669

**Published:** 2025-05-21

**Authors:** Maria-Ioanna Stefanou, Aikaterini Theodorou, Annerose Mengel, Konstantinos Melanis, Christos Bakirtzis, Vasileios Giannopapas, Dimitrios K. Kitsos, Markus Kowarik, Katharina Feil, Dimitrios Tzanetakos, Vasiliki Zouvelou, Elizabeth Andreadou, John S. Tzartos, Sotirios Giannopoulos, Georgios Tsivgoulis

**Affiliations:** Second Department of Neurology, “Attikon” University Hospital, School of Medicine, National and Kapodistrian University of Athens, Athens, Greece; Second Department of Neurology, “Attikon” University Hospital, School of Medicine, National and Kapodistrian University of Athens, Athens, Greece; Department of Neurology and Stroke, University of Tübingen, Hoppe-Seyler-Straße 3, Tübingen 72076, Germany; Hertie-Institute for Clinical Brain Research, University of Tübingen, Tübingen, Germany; Second Department of Neurology, “Attikon” University Hospital, School of Medicine, National and Kapodistrian University of Athens, Athens, Greece; Second Department of Neurology and the MS Center, AHEPA University Hospital, Central Macedonia, Thessaloniki, Greece; Second Department of Neurology, “Attikon” University Hospital, School of Medicine, National and Kapodistrian University of Athens, Athens, Greece; Second Department of Neurology, “Attikon” University Hospital, School of Medicine, National and Kapodistrian University of Athens, Athens, Greece; Department of Neurology and Stroke, University of Tübingen, Tübingen, Germany; Hertie-Institute for Clinical Brain Research, University of Tübingen, Tübingen, Germany; Department of Neurology and Stroke, University of Tübingen, Tübingen, Germany; Hertie-Institute for Clinical Brain Research, University of Tübingen, Tübingen, Germany; Second Department of Neurology, “Attikon” University Hospital, School of Medicine, National and Kapodistrian University of Athens, Athens, Greece; First Department of Neurology, “Eginition” University Hospital, School of Medicine, National and Kapodistrian University of Athens, Athens, Greece; First Department of Neurology, “Eginition” University Hospital, School of Medicine, National and Kapodistrian University of Athens, Athens, Greece; Second Department of Neurology, “Attikon” University Hospital, School of Medicine, National and Kapodistrian University of Athens, Athens, Greece; Second Department of Neurology, “Attikon” University Hospital, School of Medicine, National and Kapodistrian University of Athens, Athens, Greece; Second Department of Neurology, “Attikon” University Hospital, School of Medicine, National and Kapodistrian University of Athens, Athens, Greece

**Keywords:** cardiovascular risk factors, cerebrovascular disease, disease modifying therapies, intracerebral hemorrhage, multiple sclerosis, stroke

## Abstract

**Background:**

Epidemiological research indicates a heightened incidence of cerebrovascular disorders among patients with multiple sclerosis (MS).

**Objectives:**

The aim of the present systematic review was to investigate the potential association between disease modifying therapies (DMTs) and the risk of stroke in MS patient populations.

**Data sources and methods:**

A systematic literature search was performed in MEDLINE and SCOPUS databases up to April 6, 2024, to identify randomized-controlled clinical trials (RCT), registry-based and cohort-studies, case-series, and case-reports reporting on acute ischemic stroke (AIS) or intracerebral hemorrhage (ICH) in MS patients under different DMTs.

**Results:**

Twenty-one studies were included: 1 RCT, 6 registry-based or cohort studies, 2 case-series and 11 case-reports. Overall, DMTs appear to reduce the risk of stroke in MS patients, with DMT exposure linked to a 50% reduction of the risk of stroke compared to no DMT exposure. Although glatiramer acetate and dimethyl fumarate appear to lower the risk of stroke, concerns about fingolimod exists due to an observed elevated risk for ischemic heart disease and hypertension. Despite the absence of detected safety concerns with alemtuzumab at the MS population level, alemtuzumab-related complications, although rare, signal the need for heightened clinical vigilance. Similarly, β-interferons have been linked to life-threatening adverse events, comprising thrombotic thrombocytopenic purpura-hemolytic uremic syndrome (TTP-HUS). No associations between the risk of stroke and other DMTs, comprising natalizumab and teriflunomide, were detected; yet, newly approved DMTs were underrepresented.

**Conclusion:**

These findings highlight the importance of personalizing DMT selection and monitoring cardiovascular risk factors to reduce stroke risk in patients with MS.

**Trial registration:**

PROSPERO CRD42024534470.

## Plain language summary

Risk of stroke under disease modifying therapies for multiple sclerosis: a systematic review

Epidemiological data are sparse regarding the risk of stroke in patients with multiple sclerosis (MS). In addition, there is a paucity of evidence regarding the potential association between disease modifying therapies (DMTs) and the risk of stroke in MS patient populations. We conducted a systematic review with the aim to investigate the incidence of stroke and stroke characteristics in MS patients under different types of currently FDA (Food and Drug Administration)-approved DMTs. Our findings show that DMTs appear to reduce the risk of stroke at the MS population level, with DMT exposure linked to a 50% reduction of the risk of stroke compared to no DMT exposure. Nevertheless, this association likely varies by DMT type. Although glatiramer acetate and dimethyl fumarate appear to lower the risk of stroke, concerns regarding fingolimod have been raised, particularly regarding the observed elevated risk for ischemic heart disease and hypertension. The rare but serious complications linked to alemtuzumab and β-interferons call for rigorous monitoring in clinical practice. Finally, the methodological limitations of existing studies underscore the need for methodologically robust epidemiological research to better delineate the association between specific types of DMTs and stroke subtypes in MS, with the aim to provide a stronger evidence base for clinical decision-making.

## Introduction

Stroke and multiple sclerosis (MS) rank among the leading conditions affecting the human nervous system globally, conferring increased risk for death and disability.^1^ Recent epidemiological data disclose an exponential increase of the global prevalence of both conditions, with age-standardized prevalence estimates currently amounting to 1099 and 22 cases per 100,000 population for stroke and MS, respectively. From an epidemiological perspective, numerous studies have established that patients with MS harbor an increased risk for stroke compared to the general population^2^; yet, the pathophysiological grounds behind this association remain, to date, only partially elucidated. First, cardiovascular risk factors, including dyslipidemia, hypertension, diabetes mellitus, and metabolic syndrome, are highly prevalent among MS patients and confer increased risk for stroke in MS.^2,3^ Second, shared genetic variants have been suggested by large-scale genome-wide association studies to increase susceptibility to both conditions.^4^ Third, cardio- and neurovascular dysfunctions have been proposed to constitute direct sequelae of disease activity in MS, resulting from implication of autonomic pathways and ongoing neuroinflammatory processes in the brain.^5^

Given the intricate association between neuroinflammation and stroke, increasing attention has been drawn recently toward the potential role of disease-modifying therapies (DMTs) in relation to stroke risk among MS patients. Notably, proof-of-concept clinical trials in acute ischemic stroke (AIS) have previously investigated the neuroprotective potential of DMTs in non-MS patients with AIS.^6–9^ Despite the promising data from animal research and experimental models of AIS; however,^10–12^ these trials yielded negative results, demonstrating a lack of clinical efficacy of DMTs, including fingolimod and natalizumab, in reducing infarct volume or improving functional outcomes in AIS patients.^6–9^ In patients with MS, the so-far available data are equally conflicting. While some observational studies have suggested reduced incidence of stroke in MS patient populations under DMTs, other reports have provided contradictory findings, indicating an increased risk for major adverse cardiovascular and cerebrovascular events, including AIS and intracerebral hemorrhage (ICH) with immunomodulatory treatment.^13^ In addition, serious adverse events that precipitate stroke have been repeatedly reported in the literature, either immediately postadministration or under long-term exposure to DMTs, including hypertensive crises, metabolic imbalances, vasoconstriction, and hematological side-effects, entailing prothrombotic disorders.

Despite the acknowledged and frequent co-occurrence of stroke and MS, there is currently a critical gap in our understanding of this association in the MS patient population, as robust epidemiological evidence from MS patients under different types of DMTs is still missing.^2^ In the present systematic review, we sought to investigate the incidence of stroke among MS patients treated with different types of DMTs, while analyzing stroke characteristics from the sum of so-far published cases reporting on incident stroke under DMTs in MS patients.

## Methods

### Standard protocol approvals, registrations

The protocol for the present systematic review has been preregistered in the International Prospective Register of Ongoing Systematic Reviews PROSPERO database (CRD42024534470). The updated Preferred Reporting Items for Systematic Reviews and Meta-Analyses (PRISMA) guidelines have been employed for reporting, while reporting also adheres to the Meta-analysis of Observational Studies in Epidemiology (MOOSE) proposal.^14,15^ Ethical board approval and individual written informed consent were not required for this study as per study design (systematic review).

### Data sources, searches, and study selection

A systematic literature search was independently performed by three authors (M.-I.S., K.M., A.T.) to identify eligible studies that reported on AIS or cerebrovascular disease, including intracranial hemorrhage, in MS patients under currently Food and Drug Administration (FDA)-approved DMTs for MS. MEDLINE and SCOPUS databases were searched by applying search strings comprising the following search terms: “multiple sclerosis,” “cerebrovascular disease,” “stroke,” “intracranial hemorrhage,” “intracerebral hemorrhage,” “β-interferons,” “mitoxantrone,” “glatiramer acetate,” “teriflunomide,” “dimethyl fumarate,” “diroximel fumarate,” “monomethyl fumarate,” “fingolimod,” “siponimod,” “ozanimod,” “ponesimod,” “cladribine,” “alemtuzumab,” “natalizumab,” “ocrelizumab,” “ofatumumab,” and “ublituximab.” The full search algorithms that were used in MEDLINE and SCOPUS databases are provided in the Supplemental Material. No language or other search restrictions were applied. The search spanned from each electronic database’s inception to April 6, 2024. To ascertain the comprehensiveness of the bibliography, reference lists of published articles fulfilling our inclusion criteria were searched manually.

Clinical trials, population-based studies, or registries, along with observational studies, case-series, and case reports that reported on AIS or intracranial hemorrhage in MS patients under DMTs were eligible for inclusion. Patients were considered eligible for inclusion, provided that diagnosis was either with relapsing-remitting MS (RRMS), primary progressive MS, or secondary progressive MS. Per study protocol, studies were excluded if (1) MS or diagnoses of cerebrovascular diseases were uncertain according to our predefined inclusion criteria; (2) reported outcomes were not reported in detail or not aligned with our inclusion criteria (i.e., including studies that reported solely on cardiovascular, hematological, or other adverse effects—i.e., not including stroke); (3) they included only pediatric MS populations; (4) they were narrative and systematic reviews, commentaries, preprints or nonpeer reviewed studies, and conference abstracts. In case of data overlap between studies, we retained the study with the largest dataset. All retrieved studies were assessed by three authors (M.-I.S., A.T., K.M.) independently and any disagreements between reviewers were resolved after discussion with a fourth tie-breaking evaluator, the senior author (G.T.).

### Quality control, bias assessment, and data extraction

For relevant domains of each included study, the risk of bias was assessed using the revised Cochrane Risk of Bias tool for randomized trials (RoB 2),^16^ the Risk Of Bias In Non-randomized Studies of Interventions (ROBINS-I) tool,^17^ and the Joanna Briggs Institute Critical Appraisal Checklist for case reports and case-series.^18^ Three independent authors (M.-I.S., K.M., A.T.) performed quality control and bias assessment, and consensus after discussion with the senior author (G.T.) was reached in the case of disagreement. For further analyses, data including author names, date of publication, study design, country, event type, and patient characteristics were extracted from individual studies in structured reports.^19,20^

## Results

### Literature search and included studies

**Figure 1.**
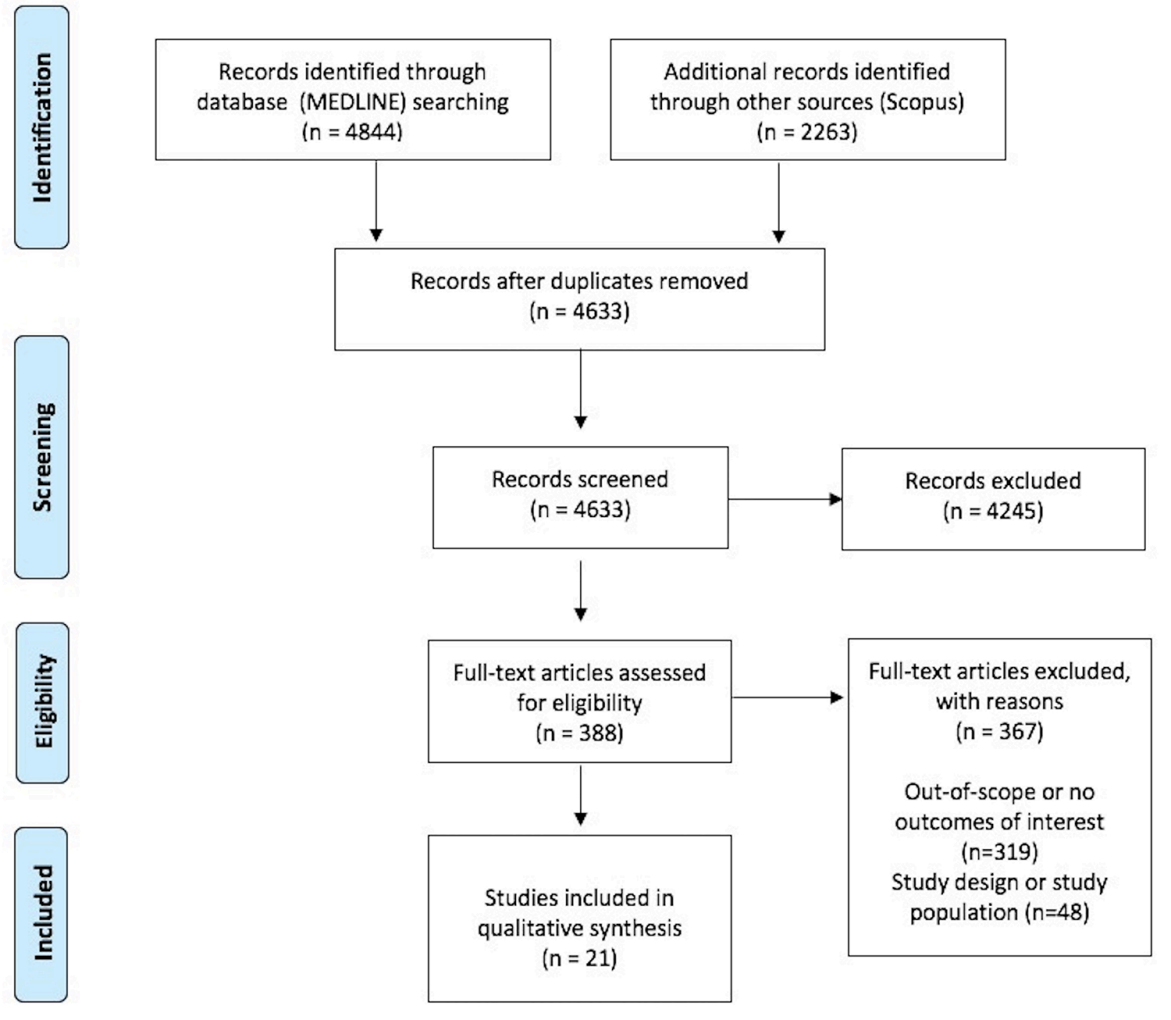
Flow chart presenting the selection of eligible studies.

The systematic database search yielded 4844 and 2263 records from MEDLINE and SCOPUS databases, respectively. After exclusion of duplicates and articles that were out-of-scope, 388 records were considered eligible for inclusion and were assessed in full. After reading the full-text articles, 367 were further excluded (Supplemental Material). Finally, we identified 21 eligible studies for inclusion, of which 1 was a randomized-controlled clinical trial (RCT; i.e., with results reported in two separate studies),^21,22^ 6 were registries or cohorts,^23–28^ 2 and 11 were case-series^29,30^ and case-reports,^31–41^ respectively. Table 1 summarizes their main characteristics, including study type, population size, and reported outcomes. In Figure 1, the PRISMA flowchart of the systematic review is presented.

**Table 1.** Overview of included studies.

Study	Type of study	Number of patients	Duration of study	DMTs	Type of MS
Simbrich et al. (2019)^23^	Retrospective, registry-based cohort based on claims data	9045	January 1, 2006–December 31, 2013	Fingolimod, natalizumab, glatiramer acetate, and IFNβ1a	All types
Ng et al. (2024)^24^	Retrospective, registry-based cohort using linked administrative health data	35,894	January 1, 1996–December 31, 2017	β-Interferon, glatiramer acetate, natalizumab, fingolimod, dimethyl fumarate, teriflunomide, and alemtuzumab	All types
Coles et al. (2008)^22^; Cuker et al. (2011)^21^	Randomized, blinded, phase II trial (CAMMS223; NCT00050778)	334	12/2002–7/2004	Alemtuzumab vs IFNβ1a	RRMS
Holmøy et al. (2019)^25^	Pharmacovigilance study	1	Until November 19, 2018	Alemtuzumab	RRMS
Azevedo et al. (2019)^29^	Case series	5	March, 2016–October, 2017	Alemtuzumab	RRMS
Durand-Dubief et al. (2019)^31^	Case report	1	–	Alemtuzumab	RRMS
Alnahdi et al. (2020)^32^	Case report	1	–	Alemtuzumab	RRMS
Inci et al. (2021)^33^	Case report	1	–	Alemtuzumab	RRMS
de Jong et al. (2017)^26^	Retrospective cohort	2485	July 1, 1995–December 31, 2004	IFN-β	RRMS
Sabidó et al. (2018)^27^	Analysis of pooled clinical trials and postmarketing surveillance	4412	For postmarketing surveillance: May 3, 1998–May 22, 2017	IFNβ1a	All types
Larochelle et al. (2014)^30^	Case series	3	2009–2012	IFNβ1a	RRMS
Niederwieser et al. (2001)^34^	Case report	1	–	IFNβ1b	RRMS
Strohm et al. (2016)^35^	Case report	1	–	IFNβ1a	RRMS
Sánchez-Soblechero et al. (2021)^36^	Case report	1	–	IFNβ1b and IFNβ1a	RRMS
Framke et al. (2024)^28^	Registry-based study	2095	2011–2018	Fingolimod Natalizumab	RRMS
Belliston et al. (2016)^37^	Case report	1	–	Fingolimod	RRMS
Fukai et al. (2019)^38^	Case report	1	–	Fingolimod	RRMS
Stich et al. (2009)^39^	Case report	1	–	Natalizumab	RRMS
de Falco et al. (2020)^40^	Case report	1	–	Teriflunomide	RRMS
Ketabforoush et al. (2023)^41^	Case report	1	–	Teriflunomide	RRMS

DMTs, disease-modifying treatments; IFNβ, interferon beta; MS, multiple sclerosis; RRMS, relapsing-remitting multiple sclerosis.

#### Quality control of included studies

**Figure 2.**
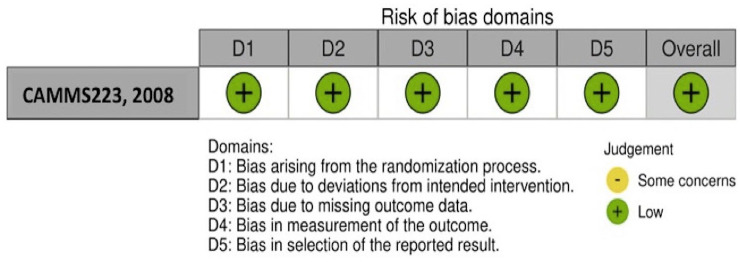
Traffic light plot presenting the quality assessment of the single included RCT using the RoB 2 tool. RCT, randomized-controlled clinical trials; RoB 2, risk of bias in randomized trials.

**Figure 3.**
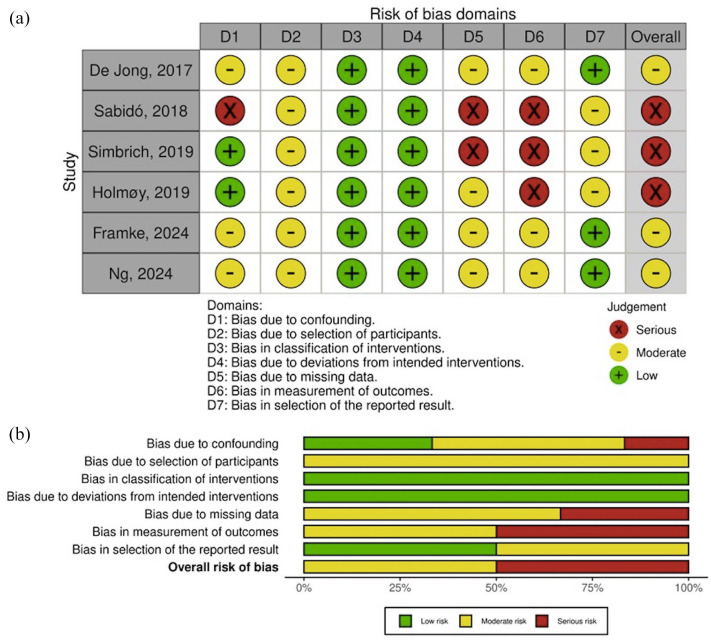
Traffic light plot (a) and bar chart (b) presenting the quality assessment of included observational studies using the ROBINS-I tool. ROBINS-I, Risk of Bias in Non-randomized Studies of Interventions.

The risk of bias of the single included RCT^21,22^ was assessed as low using the RoB 2 tool^16^ (Figure 2), while the risk of bias assessment using the ROBINS-I tool^17^ indicated moderate overall quality of included observational studies (Figure 3).^23–28^ Conversely, the risk of bias assessment of included case series^29,30^ and case reports^31–41^ using the Joanna Briggs Institute Critical Appraisal Checklist^18^ indicated an overall score of 96 of 104, which is considered to be indicative of satisfying quality (Table 2).

**Table 2.** Quality assessment of included case series and case reports using the Joanna Briggs Institute Critical Appraisal Checklist.

Study	Year	Demographics characteristics	Patient history	Current clinical condition	Assessment	Intervention	Post-intervention	Adverse events	Takeaway lessons	Overall score^a^
Niederwieser et al. (2001)^34^	2001	1	1	1	1	1	1	0	1	7
Stich et al. (2009)^39^	2009	1	0	1	1	1	1	1	1	7
Larochelle et al. (2014)^30^	2014	1	1	1	1	1	1	1	1	8
Belliston et al. (2017)^37^	2016	1	1	1	1	1	1	0	1	7
Strohm et al. (2016)^35^	2016	1	1	1	1	1	1	0	1	7
Azevedo et al. (2019)^29^	2019	1	1	1	1	1	1	0	1	7
Durand-Dubief et al. (2020)^31^	2019	1	1	1	1	1	1	0	1	7
Fukai et al. (2019)^38^	2019	1	1	1	1	1	1	0	1	7
Alnahdi et al. (2020)^32^	2020	1	1	1	1	1	1	0	1	7
De Falco et al. (2020)^40^	2020	1	1	1	1	1	1	1	1	8
Sánchez-Soblechero et al. (2022)^36^	2021	1	1	1	1	1	1	1	1	8
Inci et al. (2022)^33^	2022	1	1	1	1	1	1	1	1	8
Ketabforoush et al. (2024)^41^	2023	1	1	1	1	1	1	1	1	8
Overall score	–	13	12	13	13	13	13	6	13	96/104

a All studies presented thoroughly the patients’ demographics, discussed the current clinical condition and the assessement for their cases, and provided the diagnostic or therapeutic interventions used in their cases. The postintervention course and the takeaway lessons were discussed thoroughly in all cases as well. However, one study did not present the previous patient history^39^ and concomitant diseases and adverse events were not discussed by seven studies as well.^29,31,32,34,35,37,38^

#### Risk of stroke under different DMTs in MS patient populations

Overall, two observational studies^23,24^ reported on the risk of stroke under different types of DMTs in MS patient populations (Tables 1 and 3). The first was a registry study analyzing data nested within the German Pharmacoepidemiological Research Database (GePaRD) between January 1, 2006 and December 31, 2013.^23^ Adverse events were analyzed in a cohort of MS patients following induction of disease-modifying therapy either with fingolimod, natalizumab, glatiramer acetate, or IFNβ1a. Crude incidence rates (IRs) for adverse events were calculated per 100,000 person-years along with their corresponding 95% confidence intervals (CIs). The cohort included 9045 new DMT users, of whom 56.7% were newly assigned to IFNβ1a treatment, 38.3% to glatiramer acetate, 3.6% to natalizumab, and 1.4% to fingolimod. Stroke was classified using ICD-10 codes to encompass the diagnoses of subarachnoid hemorrhage (ICD-10: I60), intracerebral hemorrhage (ICD-10: I61), other nontraumatic intracranial hemorrhage (ICD-10: I62), cerebral infarction (ICD-10: I63) and stroke, not specified as hemorrhage or infarction (ICD-10: I64). Overall, recorded stroke events amounted to zero cases among 314 and 128 patients treated with natalizumab and fingolimod, respectively, while 5 and 3 stroke cases were recorded among 5049 and 3417 MS patients treated with IFNβ1a and glatiramer acetate, respectively. The corresponding IRs for stroke were 91.4 per 100,000 person-years for IFNβ1a (95% CI: 29.7–213.3) and 51.4 per 100,000 person-years for glatiramer acetate (95% CI: 10.6–150.2). Based on the low absolute number of observed events, the authors concluded that these estimates are aligned with the background incidence of stroke in the general population.^23^

**Table 3.** Overview of patient demographics, disease modifying therapies, and associated stroke incidents.

Study	DMTs	Gender/age	Duration of DMTs	Underlying stroke mechanism	Type of stroke	Number of stroke incidents	Treatment	Outcome
Simbrich et al. (2019)^23^	IFNβ1a Glatiramer acetate	N/A	N/A	N/A	All-cause stroke	8	N/A	N/A
Ng et al. (2024)^24^	Fingolimod	N/A	N/A	Increased hazard for ischemic heart disease and hypertension	All-cause stroke	18	N/A	N/A
Coles et al. (2008)^22^; Cuker et al. (2011)^21^	Alemtuzumab	N/A	19 months (7 months after the second cycle)	Immune thrombocytopenic purpura	ICH	1 (alemtuzumab 24 mg per day arm)	None	Fatal
Holmøy et al. (2019)^25^	Alemtuzumab	F	5 days	Autopsy revealed necrotizing vasculopathy	ICH	1	N/A	Fatal
Azevedo et al. (2019)^29^	Alemtuzumab	All five F/38–49 years	5 days	Hypertension	ICH	5	Two patients had intraventricular extension and one patient required decompressive hemicraniectomy and external ventricular drain placement	Two recovered without residual deficits, whereas three had significant neurological sequelae
Durand-Dubief et al. (2019)^31^	Alemtuzumab	F/40	4 days	Multiple cervical artery dissections	AIS in left MCA territory	1	Intravenous heparin and antiplatelet therapy	Hemiplegic and aphasic, but alive at 3-year follow-up
Alnahdi et al. (2020)^32^	Alemtuzumab	M/52	8 months	Association with alemtuzumab postulated, due to concomitant hemolytic anemia, alveolar hemorrhage, and nephropathy	AIS in bilateral occipital territories	1	Methylprednisolone, PLEX, intermittent hemodialysis, and aspirin	End-stage-kidney failure, but alive at 4-month follow-up
Inci et al. (2021)^33^	Alemtuzumab	F/52	3 days of second cycle	Possibly hypertensive	Recurrent ICH	2	Hematoma evacuation with craniotomy and external ventricular drainage placement	Fatal
de Jong et al. (2017)^26^	IFNβ	N/A	13 patients ⩽2 years; 25 patients >2 years	N/A	All-cause stroke	38	N/A	N/A
Sabidó et al. (2018)^27^	IFNβ-1a	N/A	9 patients <2 years; 16 patients ⩾2 years	N/A	All-cause stroke	15	N/A	N/A
Larochelle et al. (2014)^30^	IFNβ-1a	All 3 F/34–47 years	14–132 months	TTP-HUS	AIS and PRES	1	PLEX, corticosteroids, vincristine, and rituximab	Fatal
Niederwieser et al. (2001)^34^	IFNβ-1b	F/42	5 years	N/A	ICH	1	Craniotomy and placement of ventriculo-peritoneal shunt	Survived, but in apallic state
Strohm et al. (2016)^35^	IFNβ-1a	F/42	2 months	RCVS	SAH	1	Methylprednisolone and verapamil	Resolution of RCVS at 1-month follow-up
Sánchez-Soblechero et al. (2021)^36^	IFNβ-1b and IFNβ-1a	F/56	8 years	N/A	Recurrent ICH	1	Urgent left frontal craniotomy	Alive but with neurological deficits at 6-month follow-up
Framke et al. (2024)^28^	Fingolimod Natalizumab	N/A	N/A	N/A	All-cause stroke	<12	N/A	N/A
Belliston et al. (2016)^37^	Fingolimod	F/39	5 months	RCVS	Lobar ICH-SAH-punctuate multifocal ischemic lesions	1	Magnesium in lieu of calcium channel blockers due to continued hypotension	Resolution of RCVS at 3-week follow-up
Fukai et al. (2019)^38^	Fingolimod	F/27	10 months	N/A	Nontraumatic acute epidural hematoma	1	Neurosurgical hematoma evacuation; switch to dimethyl fumarate	No recurrence of hematoma at 1-year follow-up
Stich et al. (2009)^39^	Natalizumab	F/41	3 months	N/A	ICH	1	Withdrawal of natalizumab	Improved with neurorehabilitation
de Falco et al. (2020)^40^	Teriflunomide	M/47	1 year	N/A	Thalamic infarct	1	Intravenous alteplase (0.9 mg/kg)	Thrombolysis complicated by parenchymal hematoma; clinical improvement
Ketabforoush et al. (2023)^41^	Teriflunomide	F/48	35 days	N/A	Lacunar infarction	1	Antiplatelets and atorvastatin	Clinical improvement at 2-month follow-up

AIS, acute ischemic stroke; DMTs, disease-modifying treatments; F, female; ICH, intracerebral hemorrhage; IFNβ, interferon beta; M, male; MCA, middle cerebral artery; N/A, not available; PLEX, plasma exchange therapy; PRES, posterior reversible encephalopathy syndrome; RCVS, reversible cerebral vasoconstriction syndrome; SAH, subarachnoid hemorrhage; TTP-HUS, thrombotic thrombocytopenic purpura-hemolytic uremic syndrome.

The second study examined associations between DMT exposure and potential adverse events in a population-based study using linked administrative health data from four Canadian provinces.^24^ MS cases were followed from the first or most recent disease event on January 1, 1996, until the earliest of death, emigration, or December 31, 2017. DMT exposure comprised β-interferon, glatiramer acetate, natalizumab, fingolimod, dimethyl fumarate, teriflunomide, and alemtuzumab. Pooled estimates were calculated for cerebrovascular diseases, which were defined in accordance with ICD-10 codes to encompass: transient cerebral ischemic attacks and related syndromes (ICD-10: G45), vascular syndromes of brain in cerebrovascular diseases (ICD-10: G46), transient retinal artery occlusion (ICD-10: H34.0), and cerebrovascular diseases (ICD-10: I60-I69). From a total population of 35,894 MS patients, hazard ratios (HR) for incident adverse events were calculated (i.e., in patients exposed to different DMTs vs nonexposed patients) using stratified multivariate Cox proportional hazard models. Crude and adjusted HR estimates were reported; in the latter case after adjustment for sex, socioeconomic status, age, and individual comorbidity status of MS patients.

Compared to no DMT exposure, exposure to any DMT was associated with lower risk of cerebrovascular diseases (crude HR: 0.53, 95% CI: 0.46–0.61; adjusted HR: 0.83, 95% CI: 0.75–0.92). For different types of DMTs, β-interferon (crude HR: 0.60, 95% CI: 0.44–0.80), glatiramer acetate (crude HR: 0.55, 95% CI: 0.46–0.66), and dimethyl fumarate (crude HR: 0.34, 95% CI: 0.17–0.65) were associated with reduced risk for cerebrovascular diseases, while these associations remained significant after adjustment for glatiramer acetate (adjusted HR: 0.78, 95% CI: 0.65–0.94) and dimethyl fumarate (adjusted HR: 0.43, 95% CI: 0.22–0.84). Contrarily, fingolimod was associated with increased risk for cerebrovascular diseases (adjusted HR: 2.04, 95% CI: 1.27–3.30), while it is noteworthy that fingolimod was also associated with higher hazards of ischemic heart disease (adjusted HR: 1.64; 95% CI: 1.10–2.44) and hypertension (adjusted HR: 1.73; 95% CI: 1.30–2.31). No associations between the risk for cerebrovascular disease and exposure to natalizumab, teriflunomide, or alemtuzumab were detected. The authors commented that although findings reached statistical significance for incident adverse events under some DMTs, particularly for newly approved DMTs (i.e., alemtuzumab), the sample size may have been inadequate to detect rare adverse events.^24^

#### Risk of stroke under alemtuzumab

Two studies were retrieved that reported on safety outcomes of the CAMMS223 trial (ClinicalTrials.gov number, NCT00050778),^21,22^ a phase II randomized, blinded trial that included 334 previously untreated patients with early RRMS, assigned to receive either subcutaneous interferon beta-1a (IFNβ1a; at a dose of 44 μg) three times per week or annual intravenous cycles of alemtuzumab (at a dose of either 12 or 24 mg per day) for 36 months. Among the 334 randomized patients, 111 were assigned to subcutaneous IFNβ1a three times weekly, and 223 to annual cycles of alemtuzumab (113 receiving 12 mg per day and 110 receiving 24 mg per day). In the alemtuzumab arm, one female patient developed a fatal ICH attributed to immune thrombocytopenic purpura 19 months after the initial alemtuzumab administration and 7 months after the second alemtuzumab cycle.

In a pharmacovigilance study^25^ analyzing data retrieved from the centralized European Medicines Agency database of suspected adverse reactions related to medicinal products (EudraVigilance) up to November 19, 2018, a total of nine fatal adverse events associated with treatment with alemtuzumab were identified. Among them was a previously unreported case of fatal ICH in a female patient that developed cytokine storm and hypertension and died 5 days after receiving the first alemtuzumab infusion. Brain autopsy revealed necrotizing vasculopathy, while causality of association with alemtuzumab was deemed probable as the patient had also received the antithrombotic drug certoparin sodium.

A case-series^29^ and three case reports^31–33^ were retrieved reporting on AIS and ICH in two and eight alemtuzumab-treated patients, respectively. All clinical and stroke characteristics are summarized in Tables 1 and 3. Briefly, all patients were women, aged between 39 and 52 years. All incident events were reported within days from alemtuzumab administration, with the exception of 1 ICH^21,22^ and 1 AIS case^32^ that occurred within 7–8 months following alemtuzumab treatment. Distinct pathophysiological mechanisms were implicated, including hypertensive ICH etiology and AIS due to multiple cervical artery dissections shortly postadministration. By contrast, immune-mediated etiology was suspected in events occurring several months postadministration, including immune thrombocytopenic purpura in an ICH case,^21,22^ and suspected overt immunoreaction in an AIS case with concomitant hemolytic anemia, alveolar hemorrhage, and nephropathy.^32^ The majority of patients survived, but residual disability was reported in most cases.

#### Risk of stroke under β-interferons

Two registry-based observational studies^26,27^ examined the incidence of stroke under β-interferon therapy in the RRMS patient population. The first^26^ was a registry based and nested case–control study that reported that among RRMS patients those with incident stroke were almost twice as likely (adjusted odds ratio: 1.83, 95% CI: 1.16–2.89) to have previous exposure to IFN-b than controls (after adjustment for age). In this study, two issues are worth noting: (a) stroke was defined per ICD-10 system to encompass diagnoses ICD-10 of: I60–I69; and (b) the same group of researchers^24^ could not replicate these findings in subsequent research, demonstrating lack of association (or even a marginally negative association in unadjusted analyses) between IFN-b and the risk of stroke in a recent and significantly larger study with longer follow-up, as detailed previously.

A second^27^ investigator-sponsored analysis of clinical trials and postmarketing surveillance data provided incidence estimates of stroke from a pooled analysis of 27 clinical trials, including data from 4412 patients treated with IFN-b1a and a pooled analysis from the investigator-initiated global safety database, including a total of 2039 reported adverse events from database launch until May 3, 2017. In this analysis, the IR of stroke and IR ratio (IRR) per 100 patient-years (PY) with corresponding 95% CIs were calculated. Stroke events were retrieved based on analysis of standardized Medical Dictionary for Regulatory Activities (MedDRA) queries, comprising terms such as ischemic central nervous system vascular conditions; hemorrhagic central nervous system vascular conditions; central nervous system vascular disorders, not specified as hemorrhagic or ischemic; conditions associated with central nervous system hemorrhagic and cerebrovascular accidents. Results from these analyses demonstrated a trend toward decreased risk of stroke for IFN-b1a compared to placebo with an IRR for IFN-b1a of 0.486 (95% CI: 0.238–0.995), while the overall reporting rate of stroke was 13.286 per 10,000 PY. The researchers concluded that the safety data both from clinical trials and postmarketing settings indicate that treatment with IFN-b1a is not associated with increased risk of stroke in MS patients.^27^

A case-series^30^ and three case reports^34–36^ that reported on stroke under β-interferon treatment were also retrieved from the systematic literature search. The case-series^30^ reported on three women with RRMS, aged between 34 and 47 years, treated with IFNβ1a (44 μg 3× weekly), who were diagnosed with thrombotic thrombocytopenic purpura-hemolytic uremic syndrome (TTP-HUS) within 14–132 months of treatment. Among them, two patients developed a posterior reversible encephalopathy syndrome, with one of them presenting acute ischemic lesions on follow-up brain computed tomography scan following the resolution of the cerebral edema. This patient expired despite combined treatment with plasma exchange (PLEX), corticosteroids, vincristine, and rituximab, due to hemorrhagic shock. Notably, all three patients presented with hemolytic anemia, thrombocytopenia, and arterial hypertension, and progressed toward severe renal insufficiency despite treatment. The three case reports described two cases^34,36^ of ICH (among them one with recurrent ICH under IFN-1b and IFN-1a treatment)^36^ with unspecified underlying etiology, and one case of subarachnoid hemorrhage 2 months after initiation of IFNb-1a treatment, which was associated with reversible cerebral vasoconstriction syndrome (RCVS).^35^

#### Risk of stroke under fingolimod

One registry-based observational study^28^ examined the incidence of stroke under fingolimod therapy in the RRMS patient population. This was a nationwide 12-year cohort study that linked individual-level data from the Danish Multiple Sclerosis Registry with health registries on 2095 adults with RRMS without previous history of cardiovascular disease (CVD). The researchers investigated the relative risk of CVD, defined as the composite of ischemic heart disease, atrial fibrillation, heart failure, arterial hypertension, and stroke in fingolimod versus natalizumab-treated patients. In this study, stroke was defined to encompass the ICD-10 diagnoses I61 and I63-64. Compared to natalizumab, fingolimod was associated with an increased risk of CVD (HR = 1.57; 95% CI: 1.18–2.08). Nevertheless, the rates of stroke were comparable between fingolimod and natalizumab-treated patients. The researchers concluded that the increased risk of CVD was mainly attributed to an increased risk of hypertension in fingolimod-treated patients. Two further case reports^37,38^ were retrieved: the first^38^ reported on a patient presenting with nontraumatic acute epidural hematoma 10 months after fingolimod initiation and the second on a patient presenting with lobar ICH and subarachnoid hemorrhage due to RCVS.^37^ Detailed patient characteristics and outcomes are summarized in Table 3.

#### Risk of stroke under teriflunomide and natalizumab

Two case-reports^40,41^ describing two patients with RRMS who presented with AIS under teriflunomide treatment were retrieved (i.e., one case 1 month^41^ after treatment initiation and another^40^ after 1 year on teriflunomide treatment). In one of these cases,^40^ the patient underwent intravenous thrombolysis (IVT) with alteplase and although a small parenchymal hematoma was observed post-IVT, the patient presented significant clinical improvement. In none of these cases, a causal association between teriflunomide and AIS could be ascertained.

One case report^39^ described a patient with RRMS who presented with ICH 3 months after initiation of natalizumab treatment. The association between natalizumab and ICH was discussed; however, in this patient, cerebral autosomal-dominant arteriopathy with subcortical infarcts and leukoencephalopathy was also disclosed by skin biopsy; thus, no reliable inferences can be drawn from this anecdotal case.

## Discussion

The present systematic review has comprehensively examined the so-far available evidence on potential associations between the currently FDA-approved DMTs and the risk of stroke in MS, integrating the most recent data from population-based research.^24^ The key findings of the present study can be summarized as follows: First, two registry-based studies investigating the risk of stroke in MS populations under different DMT were identified.^23,24^ The earlier study posited that the risk of stroke in MS populations under treatment with fingolimod, natalizumab, glatiramer acetate, and IFNβ1a is aligned with the background incidence of stroke in the general population.^23^ Conversely, the most recent study suggested that the risk of stroke may vary depending on DMT exposure.^24^ Particularly with respect to the type of DMT, this study demonstrated that glatiramer acetate and dimethyl fumarate are linked to reduced risk of stroke at population level. By contrast, fingolimod was associated with a twofold increased risk of stroke and an elevated risk of ischemic heart disease and hypertension. Among other investigated DMTs, no associations were detected between the risk of stroke and β-interferon, natalizumab, teriflunomide, or alemtuzumab. Notably, this was the largest so-far published cohort, encompassing data from 35,894 MS patients.^24^ The main finding of this study was that exposure to any DMT attenuates the risk of stroke by 50% compared to no DMT exposure.

Second, despite the fact that alemtuzumab was not associated with increased risk of stroke in the aforementioned population-based study,^24^ due to short observational periods and limited sample size of exposed MS patients, the risk of stroke may have been underestimated. Our findings indicate that stroke comprises a rare yet serious adverse event associated with alemtuzumab treatment, with cases of ICH being more frequent than AIS among alemtuzumab-treated MS patients. In accordance with identified cases in the literature, the FDA has issued a safety warning about cases of stroke associated with alemtuzumab, in response to the identification of 13 incident cases of ischemic and hemorrhagic stroke postalemtuzumab administration.^42^ It is noteworthy, that distinct pathophysiological pathways are implicated in early versus delayed incidence of stroke post-alemtuzumab exposure. In the early phase (i.e., within days from exposure), hypertensive etiology and endothelial inflammation resulting in multiple cervical artery dissections have been suggested to account for ICH and AIS, respectively. By contrast, delayed immune-mediated effects (i.e., with a latency of several months postexposure), including immune thrombocytopenic purpura and autoimmune hemolytic anemia have been implicated in late-onset ICH and AIS cases.^21,22,32,43–45^

Third, as evidenced by the analysis of safety data from registries, clinical trials, and postmarketing surveillance databases, the risk of stroke with β-interferons appears comparable to that of the general population. Nevertheless, TTP-HUS constitutes an infrequent yet life-threatening complication associated with IFNβ1a therapy.^30^ It has been hypothesized that interferons inhibit the proliferation and migration of epithelial cells, thereby preventing angiogenesis and potentially triggering thrombotic microangiopathy.^46,47^ In addition, antiphospholipid antibodies and anti-ADAMTS13 IgG synthesis, along with inhibition of vascular endothelial cell growth factor activity in renal podocytes have been implicated in renal thrombotic microangiopathy in patients treated with β-interferons.^46,47^ In clinical settings, rigorous monitoring for early diagnosis of TTP-HUS is imperative, especially in patients presenting with thrombocytopenia, microangiopathic hemolytic anemia, renal or neurological impairment, and fever. For such patients, timely initiation of immunotherapy—including treatment with corticosteroids, PLEX, rituximab, cyclophosphamide, or other immunosuppressants—is crucial to prevent fatal outcomes.^48^

Fourth, only a very few studies in the literature have reported on incident stroke under fingolimod or natalizumab treatment; a finding that echoes the results from animal research studies that indicated neuroprotective potential from the these two DMTs in rodent stroke models.^10–12^ A registry study^28^ comparing fingolimod to natalizumab, however, demonstrated that fingolimod is associated with an increased risk of CVD. Notably, as the rates of stroke were comparable between fingolimod- and natalizumab-treated patients, the researchers concluded that the increased risk of CVD was mainly attributed to the increased risk of hypertension in fingolimod-treated patients. The mechanisms by which fingolimod may induce hypertension remain poorly elucidated; however, by acting on sphingosine-1-phosphate receptors on cardiac myocytes and smooth muscle cells, fingolimod has been suggested to induce changes in vascular tone and predispose to systemic hypertension.^49,50^ In keeping with the findings of the previously mentioned population-based study^24^ that disclosed an elevated risk for ischemic heart disease and hypertension with fingolimod treatment, vigorous monitoring for CVD and hypertension is required in fingolimod-treated MS patients awaiting of further real-world data.

For other examined DMTs, comprising teriflunomide and natalizumab, no safety signals with respect to stroke risk were detected in the present analysis. It should be noted however that methodological limitations may partly account for the present findings. For example, (i) underreporting may be explained by the fact that RCTs are limited in capturing rare adverse events, including stroke; (ii) there is a dearth of evidence from postmarketing surveillance studies and population-wide registries, especially for newly approved DMTs; and (iii) inherent selection and reporting biases may have confounded the results of included observational studies. In addition, it is noteworthy that although DMTs have been shown to differentially modulate cardiovascular risk factors in MS patients and certain DMTs such as teriflunomide have been associated with hypertension,^51^ and none of the retrieved studies has accounted for cardiovascular risk factors. Similarly, although patients tend to respond differently to various DMTs, the responsiveness of DMTs has not been investigated in relation to the risk of stroke in the MS patient population. Another limitation of this study is that due to the high heterogeneity in stroke definitions and included patient populations across studies, no meta-analysis could be performed. Thus, the generalizability of the present findings is limited and well-characterized cohorts and registries (i.e., providing individual patient data) are required to allow for robust meta-analyses regarding the incidence of different stroke subtypes in MS patient populations under different DMTs, while accounting for confounding factors such as preexisting cardiovascular comorbidities.

This systematic review addressed the risk of stroke under currently FDA-approved DMTs for MS, synthesizing data from a total of 21 included studies. Methodological strengths include the adherence to a prespecified, preregistered protocol for the systematic review, the detailed analysis of study characteristics and outcomes, along with the robust assessments of quality and bias. While this study provides a thorough overview of the existing literature, highlighting critical aspects for clinical practice and identifying research gaps, the limited data from RCTs or large-scale observational studies pose significant limitations. Nonetheless, while our findings primarily rely on case series and case reports, they provide essential real-world insights for the pharmacovigilance of DMTs, supporting patient monitoring and informing clinical decision-making.

Methodologically, well-selected case reports and series offer unique evidence often underrepresented in other study designs, particularly RCTs, which are frequently limited by short durations and restrictive patient criteria (e.g., excluding patients with significant cardiovascular comorbidities at increased risk of stroke). Despite inherent limitations, the quality of evidence in the included case series and reports was overall high. Although causation cannot be established through observational studies, examining potential pathophysiological mechanisms underlying an increased cardiovascular or cerebrovascular risk linked to specific DMTs is crucial. Consequently, while case reports and series may rank low within the evidence hierarchy, they may significantly contribute to clinical practice by informing DMT monitoring protocols that emphasize early detection and prevention of serious adverse events.^52,53^

## Conclusion

In conclusion, the findings of the present systematic review indicate that while FDA-approved DMTs generally appear to reduce the risk of stroke at the MS population level, this association varies by DMT type. Our findings suggest a protective role for glatiramer acetate and dimethyl fumarate and raise concerns about the risks associated with fingolimod, particularly regarding the observed elevated risk for ischemic heart disease and hypertension, although further real-world studies are required to corroborate this evidence. The rare but serious complications linked to alemtuzumab and β-interferons call for rigorous monitoring in clinical practice. Finally, the methodological limitations of existing studies underscore the need for methodologically robust epidemiological research to better delineate the association between specific types of DMTs and stroke subtypes in MS, with the aim to provide a stronger evidence base for clinical decision-making.

## Supplemental Material

sj-docx-1-tan-10.1177_17562864251321669 – Supplemental material for Risk of stroke under disease modifying therapies for multiple sclerosis: a systematic review Supplemental material, sj-docx-1-tan-10.1177_17562864251321669 for Risk of stroke under disease modifying therapies for multiple sclerosis: a systematic review by Maria-Ioanna Stefanou, Aikaterini Theodorou, Annerose Mengel, Konstantinos Melanis, Christos Bakirtzis, Vasileios Giannopapas, Dimitrios K. Kitsos, Markus Kowarik, Katharina Feil, Dimitrios Tzanetakos, Vasiliki Zouvelou, Elizabeth Andreadou, John S. Tzartos, Sotirios Giannopoulos and Georgios Tsivgoulis in Therapeutic Advances in Neurological Disorders

sj-docx-2-tan-10.1177_17562864251321669 – Supplemental material for Risk of stroke under disease modifying therapies for multiple sclerosis: a systematic review Supplemental material, sj-docx-2-tan-10.1177_17562864251321669 for Risk of stroke under disease modifying therapies for multiple sclerosis: a systematic review by Maria-Ioanna Stefanou, Aikaterini Theodorou, Annerose Mengel, Konstantinos Melanis, Christos Bakirtzis, Vasileios Giannopapas, Dimitrios K. Kitsos, Markus Kowarik, Katharina Feil, Dimitrios Tzanetakos, Vasiliki Zouvelou, Elizabeth Andreadou, John S. Tzartos, Sotirios Giannopoulos and Georgios Tsivgoulis in Therapeutic Advances in Neurological Disorders

## Declarations

**Ethics approval and consent to participate:** This study did not require an ethical board approval or written informed consent by the patients according to the study design (systematic review).

**Consent for publication:** Not applicable.

**Availability of data and materials:** The authors confirm that the data supporting the findings of this study are available within the article and its Supplemental Material.
