# Supplementary material for: Risk of stroke under disease modifying therapies for multiple sclerosis: a systematic review
Source: Ther Adv Neurol Disord. 2025 May 21;18:17562864251321669. doi: 10.1177/17562864251321669 (PMC12099087; doi:10.1177/17562864251321669)
Supplement: sj-docx-2-tan-10.1177_17562864251321669 – Supplemental material for Risk of stroke under disease modifying therapies for multiple sclerosis: a systematic review [file sj-docx-2-tan-10.1177_17562864251321669.docx]

**SUPPLEMENTAL MATERIAL**

**Complete search algorithm used in MEDLINE search.**

**Complete search algorithm used in SCOPUS search.**

**Supplementary Tables**

**Supplemental Table-S1:** Table of excluded studies with reasons for exclusion.

**ANALYSIS**

**Expanded Methods**

**Complete search algorithm used in MEDLINE search**

(Multiple sclerosis) AND ((Interferon Beta) OR (β-interferons) OR (Peginterferon Beta-1a) OR (Mitoxantrone) OR (Glatiramer Acetate) OR (Teriflunomide) OR (Dimethyl Fumarate) OR (Diroximel fumarate) OR (Monomethyl fumarate) OR (Fingolimod) OR (Siponimod) OR (Ozanimod) OR (Ponesimod) OR (Cladribine) OR (Alemtuzumab) OR (Natalizumab) OR (Ocrelizumab) OR (Ofatumumab) OR (Ublituximab)) AND ((Stroke) OR (Ischemic Stroke) OR (Hemorrhagic Stroke) OR (Hemorrhage) OR (Haemorrhage) OR (Cerebrovascular Events) OR (Adverse event) OR (Adverse events))

**Complete search algorithm used in SCOPUS search**

TITLE-ABS-KEY (( multiple AND sclerosis) AND ((interferon AND beta) OR (β-interferons) OR (peginterferon AND beta-1a) OR (mitoxantrone) OR (glatiramer AND acetate) OR (teriflunomide) OR (dimethyl AND fumarate) OR (diroximel AND fumarate) OR (monomethyl AND fumarate) OR (fingolimod) OR (siponimod) OR (ozanimod) OR (ponesimod) OR (cladribine) OR (alemtuzumab) OR (natalizumab) OR (ocrelizumab) OR (ofatumumab) OR (ublituximab)) AND ((stroke) OR (ischemic AND stroke) OR (hemorrhagic AND stroke) OR (hemorrhage) OR (haemorrhage) OR (cerebrovascular AND events) OR (adverse AND event) OR (adverse AND events)))

**Supplemental Tables**

**Supplemental Table-S1:** Table of excluded studies with reasons for exclusion (also uploaded to OSF registry and available at osf.io/v6huk).

| **Reasons for exclusion** | **PMID** | **Article title** |
| --- | --- | --- |
| No outcomes of interest | 1486115 | Treatment of multiple sclerosis with mitoxantrone |
| No outcomes of interest | 8643695 | The treatment of chronic progressive multiple sclerosis with cladribine |
| No outcomes of interest | 8714617 | Treatment of multiple sclerosis and other autoimmune diseases with cladribine |
| No outcomes of interest | 9827230 | Safety and tolerability of subcutaneous cladribine therapy in progressive multiple sclerosis |
| No outcomes of interest | 9893155 | A double-blind, placebo-controlled, randomized trial of cladribine in relapsing-remitting multiple sclerosis |
| No outcomes of interest | 9916886 | Treatment of multiple sclerosis with copolymer-1 (Copaxone): implicating mechanisms of Th1 to Th2/Th3 immune-deviation |
| Out of scope | 11502774 | Thyroid function and autoimmunity during interferon beta-1b treatment: a multicenter prospective study |
| Out of scope | 11683402 | Transmigration of PBMNCs from beta-IFN-1b-treated MS patients: a one-year longitudinal study |
| Out of scope | 11908706 | Specific proliferation towards myelin antigens in patients with multiple sclerosis during a relapse |
| No outcomes of interest | 11967257 | The immune modulator FTY720 targets sphingosine 1-phosphate receptors |
| Out of scope | 11985637 | Decrease in heart ventricular ejection fraction during multiple sclerosis |
| No outcomes of interest | 11997066 | Comparison of glatiramer acetate (Copaxone) and interferon beta-1b (Betaferon) in multiple sclerosis patients: an open-label 2-year follow-up |
| Out of scope | 12097809 | Neuropsychological performance and mood states following acute interferon-beta-1b administration in healthy males |
| Out of scope | 12412939 | Prevention of autoimmune attack and disease progression in multiple sclerosis: current therapies and future prospects |
| No outcomes of interest | 12422218 | The HMG-CoA reductase inhibitor, atorvastatin, promotes a Th2 bias and reverses paralysis in central nervous system autoimmune disease |
| No outcomes of interest | 12446022 | The macrophage activity marker sCD14 is increased in patients with multiple sclerosis and upregulated by interferon beta-1b |
| Νot population of interest | 12504397 | Mitoxantrone in progressive multiple sclerosis: a placebo-controlled, double-blind, randomised, multicentre trial |
| Study type | 12749757 | Spotlight on glatiramer acetate in relapsing-remitting multiple sclerosis |
| Οut of scope | 12796827 | Prevalence of autoimmune thyroiditis and non-immune thyroid disease in multiple sclerosis |
| Οut of scope | 12814715 | TNF-related apoptosis inducing ligand (TRAIL) as a potential response marker for interferon-beta treatment in multiple sclerosis |
| No outcomes of interest | 12926838 | Oral interferon beta-1a in relapsing-remitting multiple sclerosis: a double-blind randomized study |
| Study type | 12941579 | New and emerging treatment options for multiple sclerosis |
| No outcomes of interest | 14582768 | Interferon beta in relapsing-remitting multiple sclerosis: an independent postmarketing study in southern Italy |
| No outcomes of interest | 14716530 | Interferon beta treatment of MS in the daily clinical setting: a 3-year post-marketing study |
| No outcomes of interest | 15222692 | Treatment response in relation to inflammatory and axonal surrogate marker in multiple sclerosis |
| Οut of scope | 15261565 | Preliminary analysis of a trial of pulse cyclophosphamide in IFN-beta-resistant active MS |
| Study type | 15377176 | Spotlight on Interferon-beta-1b in relapsing-remitting and secondary progressive multiple sclerosis |
| No outcomes of interest | 15471366 | An open-label safety and drug interaction study of natalizumab (Antegren) in combination with interferon-beta (Avonex) in patients with multiple sclerosis |
| No outcomes of interest | 15471376 | A prospective study of conditions associated with multiple sclerosis in a cohort of 658 consecutive outpatients attending a multiple sclerosis clinic |
| Νot population of interest | 15623666 | Mitoxantrone for multiple sclerosis in clinical practice |
| No outcomes of interest | 15772741 | Interferon beta in relapsing-remitting multiple sclerosis. An eight years experience in a specialist multiple sclerosis centre |
| No outcomes of interest | 16053475 | The long-term safety and tolerability of high-dose interferon beta-1a in relapsing-remitting multiple sclerosis: 4-year data from the PRISMS study |
| No outcomes of interest | 16076061 | Long-term follow up of glatiramer acetate compassionate use in Belgium |
| Study type | 16207073 | Spotlight on subcutaneous recombinant interferon-beta-1a (Rebif) in relapsing-remitting multiple sclerosis |
| No outcomes of interest | 16258646 | Tolerability, adverse events and compliance to glatiramer acetate in 28 patients with multiple sclerosis using the drug continuously for at least six months |
| No outcomes of interest | 16268663 | The role of alpha-4 integrin in the aetiology of multiple sclerosis: current knowledge and therapeutic implications |
| Οut of scope | 16275091 | Neurotrophic factors in relapsing remitting and secondary progressive multiple sclerosis patients during interferon beta therapy |
| Οut of scope | 16374818 | Suppression of mitoxantrone cardiotoxicity in multiple sclerosis patients by dexrazoxane |
| No outcomes of interest | 16400839 | The use of glatiramer acetate in the treatment of multiple sclerosis |
| No outcomes of interest | 16505297 | Safety and tolerability of interferon beta-1b in pediatric multiple sclerosis |
| No outcomes of interest | 16510744 | A randomized, placebo-controlled trial of natalizumab for relapsing multiple sclerosis |
| No outcomes of interest | 16567708 | A Phase II study of the safety and efficacy of teriflunomide in multiple sclerosis with relapses |
| No outcomes of interest | 16796584 | Oral fumaric acid esters for the treatment of active multiple sclerosis: an open-label, baseline-controlled pilot study |
| No outcomes of interest | 16819420 | The natural history of optic neuritis |
| Οut of scope | 16920574 | Potential adverse events with biologic response modifiers |
| No outcomes of interest | 17229751 | Early mitoxantrone-induced cardiotoxicity in secondary progressive multiple sclerosis |
| No outcomes of interest | 17480177 | The safety and efficacy of IFN-beta products for the treatment of multiple sclerosis |
| No outcomes of interest | 17548453 | Autoimmune hemolytic anemia during interferon-beta-I b treatment for multiple sclerosis |
| No outcomes of interest | 17603763 | Treatment of early-onset multiple sclerosis with intramuscular interferonbeta-1a: long-term results |
| Νot population of interest | 17846110 | Mitoxantrone as induction treatment in aggressive relapsing remitting multiple sclerosis: treatment response factors in a 5 year follow-up observational study of 100 consecutive patients |
| No outcomes of interest | 17880754 | The potential role for cladribine in the treatment of multiple sclerosis: clinical experience and development of an oral tablet formulation |
| Οut of scope | 17884184 | TRAIL, CXCL10 and CCL2 plasma levels during long-term Interferon-beta treatment of patients with multiple sclerosis correlate with flu-like adverse effects but do not predict therapeutic response |
| No outcomes of interest | 17956447 | A longitudinal observational study of a cohort of patients with relapsing-remitting multiple sclerosis treated with glatiramer acetate |
| No outcomes of interest | 18035202 | Full results of the Evidence of Interferon Dose-Response-European North American Comparative Efficacy (EVIDENCE) study: a multicenter, randomized, assessor-blinded comparison of low-dose weekly versus high-dose, high-frequency interferon beta-1a for relapsing multiple sclerosis |
| Οut of scope | 18195670 | Treating multiple sclerosis in the natalizumab era: risks, benefits, clinical decision making, and a comparison between North American and European Union practices |
| No outcomes of interest | 18208875 | Long-term (up to 22 years), open-label, compassionate-use study of glatiramer acetate in relapsing-remitting multiple sclerosis |
| Οut of scope | 18218286 | Two recombinant human interferon-beta 1a pharmaceutical preparations produce a similar transcriptional response determined using whole genome microarray analysis |
| Οut of scope | 18272865 | Predictive markers for response to interferon therapy in patients with multiple sclerosis |
| Νot population of interest | 18383069 | Rituximab in relapsing-remitting multiple sclerosis: a 72-week, open-label, phase I trial |
| Οut of scope | 18393777 | Recent clinical trials of cladribine in hematological malignancies and autoimmune disorders |
| Study type | 18425915 | Recombinant interferon beta or glatiramer acetate for delaying conversion of the first demyelinating event to multiple sclerosis |
| No outcomes of interest | 18445781 | The function of the human interferon-beta 1a glycan determined in vivo |
| No outcomes of interest | 18640466 | Tolerability and safety profile of 12- to 28-week treatment with interferon beta-1b 250 and 500 microg QOD in patients with relapsing-remitting multiple sclerosis: a multicenter, randomized, double-blind, parallel-group pilot study |
| Study type | 18713565 | Spotlight on anti-CD20 |
| No outcomes of interest | 18825438 | The OPTimization of interferon for MS study: 375 microg interferon beta-1b in suboptimal responders |
| No outcomes of interest | 18946064 | Alemtuzumab vs. interferon beta-1a in early multiple sclerosis |
| No outcomes of interest | 18970976 | Efficacy and safety of oral fumarate in patients with relapsing-remitting multiple sclerosis: a multicentre, randomised, double-blind, placebo-controlled phase IIb study |
| Study type | 19479722 | New pieces in the puzzle: how does interferon-beta really work in multiple sclerosis? |
| Study type | 19603540 | Treating MS: getting to know the two birds in the bush |
| No outcomes of interest | 19729344 | 250 microg or 500 microg interferon beta-1b versus 20 mg glatiramer acetate in relapsing-remitting multiple sclerosis: a prospective, randomised, multicentre study |
| No outcomes of interest | 19770542 | Efficacy and safety of mitoxantrone, as an initial therapy, in multiple sclerosis: experience in an Indian tertiary care setting |
| No outcomes of interest | 19797261 | A single-center, randomized, double-blind, placebo-controlled study of interferon beta-1b on primary progressive and transitional multiple sclerosis |
| Νot population of interest | 19847908 | Rituximab in patients with primary progressive multiple sclerosis: results of a randomized double-blind placebo-controlled multicenter trial |
| No outcomes of interest | 19876786 | The role of sphingosine-1-phosphate receptor modulators in the prevention of transplant rejection and autoimmune diseases |
| No outcomes of interest | 20028707 | Phase II study of oral fingolimod (FTY720) in multiple sclerosis: 3-year results |
| No outcomes of interest | 20038760 | The mechanism of action of glatiramer acetate treatment in multiple sclerosis |
| No outcomes of interest | 20089952 | A placebo-controlled trial of oral fingolimod in relapsing multiple sclerosis |
| No outcomes of interest | 20089954 | Oral fingolimod or intramuscular interferon for relapsing multiple sclerosis |
| No outcomes of interest | 20089960 | A placebo-controlled trial of oral cladribine for relapsing multiple sclerosis |
| Οut of scope | 20138139 | Sickness behaviour is induced by a peripheral CXC-chemokine also expressed in multiple sclerosis and EAE |
| Οut of scope | 20378007 | Soluble CD30: a biomarker for evaluating the clinical risk versus benefit of IFNbeta1A treatment in multiple sclerosis patients |
| No outcomes of interest | 20426705 | Treating multiple sclerosis with fingolimod or intramuscular interferon |
| No outcomes of interest | 20427751 | Cardiotoxicity and other adverse events associated with mitoxantrone treatment for MS |
| No outcomes of interest | 20517533 | Oral fingolimod for the treatment of relapsing-remitting multiple sclerosis |
| No outcomes of interest | 20530324 | Cross-sectional study assessing long-term safety of interferon-beta-1b for relapsing-remitting MS |
| Οut of scope | 20548047 | Critical vasospasm during fingolimod (FTY720) treatment in a patient with multiple sclerosis |
| No outcomes of interest | 20556456 | Swedish natalizumab (Tysabri) multiple sclerosis surveillance study |
| Study type | 20591737 | New lessons about old molecules: how type I interferons shape Th1/Th17-mediated autoimmunity in the CNS |
| Study type | 20615054 | Recommendations for the selection, treatment, and management of patients utilizing natalizumab therapy for multiple sclerosis |
| No outcomes of interest | 20644975 | Three years of experience: the Italian registry and safety data update |
| Οut of scope | 20670985 | Preclinical studies of methylthioadenosine for the treatment of multiple sclerosis |
| Νot population of interest | 20727669 | Mitoxantrone for worsening multiple sclerosis: tolerability, toxicity, adherence and efficacy in the clinical setting |
| No outcomes of interest | 21088044 | The efficacy of natalizumab in patients with multiple sclerosis according to level of disability: results of an observational study |
| No outcomes of interest | 21160051 | The role of glycogen synthase kinase 3 in regulating IFN-β-mediated IL-10 production |
| No outcomes of interest | 21228027 | A Swedish national post-marketing surveillance study of natalizumab treatment in multiple sclerosis |
| No outcomes of interest | 21228029 | Safety and tolerability of cladribine tablets in multiple sclerosis: the CLARITY (CLAdRIbine Tablets treating multiple sclerosis orallY) study |
| No outcomes of interest | 21234710 | The strategies used for treatment of experimental autoimmune neuritis (EAN): a beneficial effect of glatiramer acetate administered intraperitoneally |
| No outcomes of interest | 21325016 | Long-term safety profile of mitoxantrone in a French cohort of 802 multiple sclerosis patients: a 5-year prospective study |
| No outcomes of interest | 21339490 | The neurobiology of sphingosine 1-phosphate signaling and sphingosine 1-phosphate receptor modulators |
| No outcomes of interest | 21387248 | An observational study of the effectiveness and safety of natalizumab in the treatment of multiple sclerosis |
| Study type | 21402415 | Recent insights into the mechanism of action of glatiramer acetate |
| No outcomes of interest | 21419858 | The Sphingosine-1 Phosphate receptor agonist FTY720 dose dependently affected endothelial integrity in vitro and aggravated ventilator-induced lung injury in mice |
| No outcomes of interest | 21491095 | Treatment with natalizumab in relapsing-remitting multiple sclerosis patients induces changes in inflammatory mechanism |
| Οut of scope | 21632839 | Increased incidence of central nervous system hemorrhages in patients with secondary acute promyelocytic leukemia after treatment of multiple sclerosis with mitoxantrone? |
| No outcomes of interest | 21649449 | The mechanism of action of interferon-β in relapsing multiple sclerosis |
| No outcomes of interest | 21718390 | Weekly IM interferon beta-1a in multiple sclerosis patients over 50 years of age |
| No outcomes of interest | 21775738 | Immune thrombocytopenic purpura in a patient with multiple sclerosis treated with natalizumab |
| No outcomes of interest | 21880336 | Efficacy and safety of subcutaneous interferon β-1a in relapsing-remitting multiple sclerosis: further outcomes from the IMPROVE study |
| No outcomes of interest | 21899662 | A single-arm, open-label study of alemtuzumab in treatment-refractory patients with multiple sclerosis |
| No outcomes of interest | 21999176 | An open-label, multicenter study to evaluate the safe and effective use of the single-use autoinjector with an Avonex® prefilled syringe in multiple sclerosis subjects |
| Οut of scope | 22022398 | Neuroprotective effect of combination therapy of glatiramer acetate and epigallocatechin-3-gallate in neuroinflammation |
| No outcomes of interest | 22046309 | Adverse events of interferon beta-1a: a prospective multi-centre international ICH-GCP-based CRO-supported external validation study in daily practice |
| No outcomes of interest | 22047971 | Ocrelizumab in relapsing-remitting multiple sclerosis: a phase 2, randomised, placebo-controlled, multicentre trial |
| No outcomes of interest | 22091593 | Treating multiple sclerosis with natalizumab |
| No outcomes of interest | 22161847 | ITP: tolerance lost |
| No outcomes of interest | 22276072 | Treatment de-escalation after mitoxantrone therapy: results of a phase IV, multicentre, open-label, randomized study of subcutaneous interferon beta-1a in patients with relapsing multiple sclerosis |
| No outcomes of interest | 22307384 | Long-term follow-up of a phase 2 study of oral teriflunomide in relapsing multiple sclerosis: safety and efficacy results up to 8.5 years |
| No outcomes of interest | 22354739 | A randomized, controlled trial of fingolimod (FTY720) in Japanese patients with multiple sclerosis |
| No outcomes of interest | 22391507 | The relationship between inflammatory activity and brain atrophy in natalizumab treated patients |
| No outcomes of interest | 22438058 | Severe haematological complications during treatment with natalizumab |
| Οut of scope | 22440894 | Therapy-related acute leukemia in two patients with multiple sclerosis treated with Mitoxantrone |
| Study type | 22573625 | The elusive biomarker for personalized medicine in multiple sclerosis: the search continues |
| Οut of scope | 22591300 | Predicting risk of progressive multifocal leukoencephalopathy from natalizumab |
| No outcomes of interest | 22596228 | Natalizumab-associated reversible encephalopathy syndrome mimicking progressive multifocal leukoencephalopathy |
| No outcomes of interest | 22615252 | The sphingosine-1-phosphate analogue FTY720 impairs mucosal immunity and clearance of the enteric pathogen Citrobacter rodentium |
| No outcomes of interest | 22622860 | Teriflunomide added to interferon-β in relapsing multiple sclerosis: a randomized phase II trial |
| Οut of scope | 22718434 | Low-dose alemtuzumab-associated immune thrombocytopenia in chronic lymphocytic leukemia |
| Οut of scope | 22967748 | Soluble thrombomodulin levels in plasma of multiple sclerosis patients and their implication |
| Οut of scope | 23026222 | Glatiramer Acetate administration does not reduce damage after cerebral ischemia in mice |
| No outcomes of interest | 23122650 | Alemtuzumab for patients with relapsing multiple sclerosis after disease-modifying therapy: a randomised controlled phase 3 trial |
| No outcomes of interest | 23122652 | Alemtuzumab versus interferon beta 1a as first-line treatment for patients with relapsing-remitting multiple sclerosis: a randomised controlled phase 3 trial |
| No outcomes of interest | 23140228 | Subcutaneous IFN-β1a to treat relapsing-remitting multiple sclerosis |
| No outcomes of interest | 23504050 | Efficacy and safety of interferon beta-1b sc in older RRMS patients--a posthoc analysis of the BEYOND study |
| No outcomes of interest | 23558379 | The outlook for alemtuzumab in multiple sclerosis |
| Study type | 23686821 | Three times weekly glatiramer acetate in relapsing-remitting multiple sclerosis |
| No outcomes of interest | 23689307 | Swiss analysis of multiple sclerosis: a multicenter, non-interventional, retrospective cohort study of disease-modifying therapies |
| Νot population of interest | 23728638 | Mitoxantrone for multiple sclerosis |
| No outcomes of interest | 23764350 | Siponimod for patients with relapsing-remitting multiple sclerosis (BOLD): an adaptive, dose-ranging, randomised, phase 2 study |
| No outcomes of interest | 23795716 | The pharmacokinetics of glatiramer acetate for multiple sclerosis treatment |
| No outcomes of interest | 23861637 | Safety and efficacy of fingolimod in treatment-naïve multiple sclerosis patients |
| No outcomes of interest | 23895407 | A prospective observational post-marketing study of natalizumab-treated multiple sclerosis patients: clinical, radiological and biological features and adverse events. The BIONAT cohort |
| Study type | 23944289 | The fumaric acid ester BG-12: a new option in MS therapy |
| Study type | 23997924 | Teriflunomide in relapsing multiple sclerosis: therapeutic utility |
| No outcomes of interest | 24119301 | Disease modifying therapies modulate cardiovascular risk factors in patients with multiple sclerosis |
| Study type | 24129036 | New insights into an autoimmune mechanism, pharmacological treatment and relationship between multiple sclerosis and inflammatory bowel disease |
| Οut of scope | 24131589 | Prevalence of cutaneous adverse events associated with long-term disease-modifying therapy and their impact on health-related quality of life in patients with multiple sclerosis: a cross-sectional study |
| Νot population of interest | 24139067 | Safety and efficacy of mitoxantrone in pediatric patients with aggressive multiple sclerosis |
| No outcomes of interest | 24139424 | Tolerability and pharmacokinetics of delayed-release dimethyl fumarate administered with and without aspirin in healthy volunteers |
| Οut of scope | 24223820 | Preserved antigen-specific immune response in patients with multiple sclerosis responding to IFNβ-therapy |
| No outcomes of interest | 24250925 | The molecular study of IFNβ pleiotropic roles in MS treatment |
| Study type | 24251808 | New and emerging immune-targeted drugs for the treatment of multiple sclerosis |
| Study type | 24321165 | Teriflunomide for the treatment of multiple sclerosis |
| Οut of scope | 24368840 | Predicting autoimmunity after alemtuzumab treatment of multiple sclerosis |
| No outcomes of interest | 24453078 | Safety and efficacy of ofatumumab in relapsing-remitting multiple sclerosis: a phase 2 study |
| Οut of scope | 24453711 | Successful management of a neurology infusion practice |
| No outcomes of interest | 24461574 | Oral teriflunomide for patients with relapsing multiple sclerosis (TOWER): a randomised, double-blind, placebo-controlled, phase 3 trial |
| No outcomes of interest | 24475777 | Fingolimod (FTY720) therapy in Japanese patients with relapsing multiple sclerosis over 12 months: results of a phase 2 observational extension |
| No outcomes of interest | 24503666 | Tip variant focal segmental glomerulosclerosis associated with interferon-β treatment of multiple sclerosis |
| No outcomes of interest | 24566807 | Switching from natalizumab to fingolimod in multiple sclerosis: a French prospective study |
| No outcomes of interest | 24659797 | Oral ponesimod in relapsing-remitting multiple sclerosis: a randomised phase II trial |
| No outcomes of interest | 24685276 | Safety and efficacy of fingolimod in patients with relapsing-remitting multiple sclerosis (FREEDOMS II): a double-blind, randomised, placebo-controlled, phase 3 trial |
| Study type | 24686106 | Recruitment of participants to a multiple sclerosis trial: the CombiRx experience |
| No outcomes of interest | 24690227 | Safety of the first dose of fingolimod for multiple sclerosis: results of an open-label clinical trial |
| Study type | 24696053 | Thrombotic thrombocytopenic purpura-haemolytic uremic syndrome in relapsing-remitting multiple sclerosis patients on high-dose interferon beta |
| Study type | 24740824 | Teriflunomide and its mechanism of action in multiple sclerosis |
| No outcomes of interest | 24788965 | Glatiramer acetate (copaxone) modulates platelet activation and inhibits thrombin-induced calcium influx: possible role of copaxone in targeting platelets during autoimmune neuroinflammation |
| No outcomes of interest | 24794721 | Pegylated interferon β-1a for relapsing-remitting multiple sclerosis (ADVANCE): a randomised, phase 3, double-blind study |
| No outcomes of interest | 24821636 | The N-terminal pro-brain natriuretic peptide as a marker of mitoxantrone-induced cardiotoxicity in multiple sclerosis patients |
| Οut of scope | 24831186 | Previous treatment influences fingolimod efficacy in relapsing-remitting multiple sclerosis: results from an observational study |
| No outcomes of interest | 24849515 | Alemtuzumab treatment of multiple sclerosis: long-term safety and efficacy |
| No outcomes of interest | 24850724 | The role of endogenous IFN-β in the regulation of Th17 responses in patients with relapsing-remitting multiple sclerosis |
| No outcomes of interest | 24855407 | Efficacy and side effects of natalizumab therapy in patients with multiple sclerosis |
| Οut of scope | 24885345 | Small non-coding RNA signature in multiple sclerosis patients after treatment with interferon-β |
| No outcomes of interest | 24898925 | Long-term safety and effectiveness of natalizumab redosing and treatment in the STRATA MS Study |
| No outcomes of interest | 24935240 | Drug safety evaluation of alemtuzumab for multiple sclerosis |
| No outcomes of interest | 24935480 | Overview and safety of fingolimod hydrochloride use in patients with multiple sclerosis |
| No outcomes of interest | 24953054 | Association between beta-interferon exposure and hospital events in multiple sclerosis |
| No outcomes of interest | 24965353 | Efficacy and safety of subcutaneous interferon-β-1a in patients with a first demyelinating event and early multiple sclerosis |
| No outcomes of interest | 24990854 | Efficacy and safety of delayed-release dimethyl fumarate in patients newly diagnosed with relapsing-remitting multiple sclerosis (RRMS) |
| Οut of scope | 25003359 | Fingolimod for the treatment of intracerebral hemorrhage: a 2-arm proof-of-concept study |
| No outcomes of interest | 25011422 | Real-world use of fingolimod in patients with relapsing remitting multiple sclerosis: a retrospective study using the national multiple sclerosis registry in Kuwait |
| No outcomes of interest | 25119836 | A 2-year observational study of patients with relapsing-remitting multiple sclerosis converting to glatiramer acetate from other disease-modifying therapies: the COPTIMIZE trial |
| No outcomes of interest | 25192851 | Oral teriflunomide for patients with a first clinical episode suggestive of multiple sclerosis (TOPIC): a randomised, double-blind, placebo-controlled, phase 3 trial |
| No outcomes of interest | 25245812 | Efficacy and safety of fingolimod in Hispanic patients with multiple sclerosis: pooled clinical trial analyses |
| No outcomes of interest | 25252236 | The real-life experience with cardiovascular complications in the first dose of fingolimod for multiple sclerosis |
| No outcomes of interest | 25342053 | The efficacy and safety of natalizumab for the treatment of multiple sclerosis in Portugal: a retrospective study |
| Οut of scope | 25398464 | Treatment of mood disorders in multiple sclerosis |
| Οut of scope | 25586466 | NLRP3 inflammasome is associated with the response to IFN-β in patients with multiple sclerosis |
| No outcomes of interest | 25687236 | Safety of teriflunomide for the management of relapsing-remitting multiple sclerosis |
| Οut of scope | 25736057 | TRAIL and TRAIL receptors splice variants during long-term interferon β treatment of patients with multiple sclerosis: evaluation as biomarkers for therapeutic response |
| No outcomes of interest | 25795646 | Long-term effects of fingolimod in multiple sclerosis: the randomized FREEDOMS extension trial |
| No outcomes of interest | 25876467 | Short-term and long-term safety and tolerability of interferon β-1b in multiple sclerosis |
| No outcomes of interest | 25877062 | Fingolimod in relapsing multiple sclerosis: An integrated analysis of safety findings |
| Οut of scope | 25977275 | Dimethyl Fumarate Protects Brain From Damage Produced by Intracerebral Hemorrhage by Mechanism Involving Nrf2 |
| No outcomes of interest | 26024899 | Switching from natalizumab to fingolimod: A randomized, placebo-controlled study in RRMS |
| Οut of scope | 26157340 | Non-Hodgkin Lymphoma of the Stomach in a Patient Treated with Natalizumab |
| No outcomes of interest | 26166235 | Tolerability and Safety of Combined Glatiramer Acetate and N-Acetylcysteine in Relapsing-Remitting Multiple Sclerosis |
| No outcomes of interest | 26170105 | Safety and Tolerability of Fingolimod in Latin American Patients with Relapsing-Remitting Multiple Sclerosis: The Open-Label FIRST LATAM Study |
| No outcomes of interest | 26198913 | Safety and efficacy of reduced fingolimod dosage treatment |
| No outcomes of interest | 26233537 | Postmarketing Safety Profile of Subcutaneous Interferon Beta-1a Given 3 Times Weekly: A Retrospective Administrative Claims Analysis |
| No outcomes of interest | 26265273 | Outcomes of switching directly to oral fingolimod from injectable therapies: Results of the randomized, open-label, multicenter, Evaluate Patient OutComes (EPOC) study in relapsing multiple sclerosis |
| No outcomes of interest | 26265275 | First-dose effects of fingolimod: Pooled safety data from three phase 3 studies |
| Οut of scope | 26300705 | Prevention and Management of Infusion-Associated Reactions in the Comparison of Alemtuzumab and Rebif(®) Efficacy in Multiple Sclerosis (CARE-MS) Program |
| No outcomes of interest | 26338810 | Long-term results from a phase 2 extension study of fingolimod at high and approved dose in relapsing multiple sclerosis |
| No outcomes of interest | 26341389 | Toxicity of teriflunomide in aryl hydrocarbon receptor deficient mice |
| No outcomes of interest | 26365096 | Teriflunomide: a once-daily oral medication for the treatment of relapsing forms of multiple sclerosis |
| No outcomes of interest | 26376649 | Treatment against human endogenous retrovirus: a possible personalized medicine approach for multiple sclerosis |
| Οut of scope | 26475926 | TNFR2 Deficiency Acts in Concert with Gut Microbiota To Precipitate Spontaneous Sex-Biased Central Nervous System Demyelinating Autoimmune Disease |
| No outcomes of interest | 26514979 | Alemtuzumab for multiple sclerosis: Long term follow-up in a multi-centre cohort |
| No outcomes of interest | 26616877 | Safety and efficacy of fingolimod in clinical practice: The experience of an academic center in the Middle East |
| No outcomes of interest | 26715860 | Clinical efficacy, safety, and tolerability of fingolimod for the treatment of relapsing-remitting multiple sclerosis |
| No outcomes of interest | 26734938 | Efficacy and Safety of Fingolimod in an Unselected Patient Population |
| Study type | 26758290 | Teriflunomide in Patients with Relapsing-Remitting Forms of Multiple Sclerosis |
| No outcomes of interest | 26818160 | The use of immune modulating drugs for the treatment of multiple sclerosis |
| No outcomes of interest | 26827074 | Oral fingolimod in primary progressive multiple sclerosis (INFORMS): a phase 3, randomised, double-blind, placebo-controlled trial |
| No outcomes of interest | 26849839 | Comparison of LC-UV and LC-MS methods for simultaneous determination of teriflunomide, dimethyl fumarate and fampridine in human plasma: application to rat pharmacokinetic study |
| No outcomes of interest | 26856942 | The management and outcomes of fingolimod first dose cardiac monitoring in UK patients with relapsing-remitting multiple sclerosis |
| No outcomes of interest | 26856952 | Pooled safety and tolerability data from four placebo-controlled teriflunomide studies and extensions |
| No outcomes of interest | 26865517 | Long-term safety and efficacy of teriflunomide: Nine-year follow-up of the randomized TEMSO study |
| No outcomes of interest | 26879276 | Safety and efficacy of the selective sphingosine 1-phosphate receptor modulator ozanimod in relapsing multiple sclerosis (RADIANCE): a randomised, placebo-controlled, phase 2 trial |
| Οut of scope | 26900439 | Effect of Fingolimod on Platelet Count Among Multiple Sclerosis Patients |
| No outcomes of interest | 26980848 | Simultaneous early-onset immune thrombocytopenia and autoimmune thyroid disease following alemtuzumab treatment in relapsing-remitting multiple sclerosis |
| No outcomes of interest | 27001955 | Topical Application of Fingolimod Perturbs Cutaneous Inflammation |
| No outcomes of interest | 27012659 | Reversible cerebral vasoconstriction syndrome associated with interferon beta-1a use for multiple sclerosis |
| No outcomes of interest | 27032105 | The Swiss Multiple Sclerosis Cohort-Study (SMSC): A Prospective Swiss Wide Investigation of Key Phases in Disease Evolution and New Treatment Options |
| Νot population of interest | 27038238 | Rituximab versus fingolimod after natalizumab in multiple sclerosis patients |
| Οut of scope | 27075495 | Precision medicine in multiple sclerosis: biomarkers for diagnosis, prognosis, and treatment response |
| Οut of scope | 27135594 | Rebound Syndrome in Patients With Multiple Sclerosis After Cessation of Fingolimod Treatment |
| Οut of scope | 27174529 | Selective Sphingosine-1-Phosphate Receptor 1 Modulation Attenuates Experimental Intracerebral Hemorrhage |
| No outcomes of interest | 27207449 | Long-term effects of delayed-release dimethyl fumarate in multiple sclerosis: Interim analysis of ENDORSE, a randomized extension study |
| No outcomes of interest | 27237769 | Long-term efficacy and safety of intramuscular interferon beta-1a: Randomized postmarketing trial of two dosing regimens in Japanese patients with relapsing-remitting multiple sclerosis |
| No outcomes of interest | 27252601 | Safety and Tolerability of Delayed-Release Dimethyl Fumarate Administered with Interferon Beta or Glatiramer Acetate in Relapsing-Remitting Multiple Sclerosis |
| No outcomes of interest | 27380540 | Safety and Efficacy of Siponimod (BAF312) in Patients With Relapsing-Remitting Multiple Sclerosis: Dose-Blinded, Randomized Extension of the Phase 2 BOLD Study |
| No outcomes of interest | 27388874 | Effect of Fingolimod-Treatment on Blood Lipid Profiles of Multiple Sclerosis Patients |
| Οut of scope | 27434365 | Tp-Te interval predicts heart rate reduction after fingolimod administration in patients with multiple sclerosis |
| Οut of scope | 27456870 | Update on the cardiovascular profile of fingolimod in the therapy of relapsing-remitting multiple sclerosis (MS) |
| No outcomes of interest | 27503905 | Efficacy and safety of a three-times-weekly dosing regimen of glatiramer acetate in relapsing-remitting multiple sclerosis patients: 3-year results of the Glatiramer Acetate Low-Frequency Administration open-label extension study |
| Οut of scope | 27516414 | Nicolau syndrome and localized panniculitis: a report of dual diagnoses with an emphasis on morphea profunda-like changes following injection with glatiramer acetate |
| No outcomes of interest | 27624575 | Cardiac Safety Profile of First Dose of Fingolimod for Relapsing-Remitting Multiple Sclerosis in Real-World Settings: Data from a German Prospective Multi-Center Observational Study |
| Οut of scope | 27653757 | Predictors of Response to Multiple Sclerosis Therapeutics in Individual Patients |
| No outcomes of interest | 27757552 | Safety and tolerability of fingolimod in patients with relapsing-remitting multiple sclerosis: results of an open-label clinical trial in Italy |
| Νot population of interest | 27760868 | Rituximab in multiple sclerosis: A retrospective observational study on safety and efficacy |
| No outcomes of interest | 27785735 | Real-World Outcomes in Fingolimod-Treated Patients with Multiple Sclerosis in the Czech Republic: Results from the 12-Month GOLEMS Study |
| No outcomes of interest | 27819760 | Reversible cerebral vasoconstriction syndrome in association with fingolimod use |
| No outcomes of interest | 27829656 | Safety and Efficacy of Fingolimod and Natalizumab in Multiple Sclerosis After the Failure of First-Line Therapy: Single Center Experience Based on the Treatment of Forty-Four Patients |
| No outcomes of interest | 27921221 | Safety and Efficacy of Natalizumab in Japanese Patients with Relapsing-Remitting Multiple Sclerosis: Open-Label Extension Study of a Phase 2 Trial |
| No outcomes of interest | 27989217 | Long-term safety and tolerability of glatiramer acetate 20 mg/ml in the treatment of relapsing forms of multiple sclerosis |
| No outcomes of interest | 28002679 | Ocrelizumab versus Interferon Beta-1a in Relapsing Multiple Sclerosis |
| No outcomes of interest | 28002688 | Ocrelizumab versus Placebo in Primary Progressive Multiple Sclerosis |
| No outcomes of interest | 28071330 | Subcutaneous peginterferon β-1a injection-site reaction experience and mitigation: Delphi analysis of the ALLOW study |
| Οut of scope | 28109694 | SIRT1 as a potential biomarker of response to treatment with glatiramer acetate in multiple sclerosis |
| No outcomes of interest | 28129749 | Long-term efficacy and safety of fingolimod in Japanese patients with relapsing multiple sclerosis: 3-year results of the phase 2 extension study |
| Οut of scope | 28180112 | Oral Multiple Sclerosis Drugs Inhibit the In vitro Growth of Epsilon Toxin Producing Gut Bacterium, Clostridium perfringens |
| No outcomes of interest | 28218904 | The protumorigenic potential of FTY720 by promoting extramedullary hematopoiesis and MDSC accumulation |
| No outcomes of interest | 28282574 | Long-term effectiveness and safety of natalizumab in a Portuguese population |
| No outcomes of interest | 28287030 | Immediate transient thrombocytopenia at the time of alemtuzumab infusion in multiple sclerosis |
| Study type | 28321835 | Teriflunomide for multiple sclerosis in real-world setting |
| Νot population of interest | 28389054 | The Use of Natalizumab in Pediatric Patients With Active Relapsing Multiple Sclerosis: A Prospective Study |
| No outcomes of interest | 28440858 | Treatment with disease-modifying drugs for people with a first clinical attack suggestive of multiple sclerosis |
| No outcomes of interest | 28453541 | The real-world effectiveness and safety of fingolimod in relapsing-remitting multiple sclerosis patients: An observational study |
| No outcomes of interest | 28485678 | The majority of natalizumab-treated MS patients have high natalizumab concentrations at time of re-dosing |
| No outcomes of interest | 28500224 | Evaluating the safety of β-interferons in MS: A series of nested case-control studies |
| Οut of scope | 28507603 | Fingolimod initiation in multiple sclerosis patients is associated with potential beneficial cardiovascular autonomic effects |
| No outcomes of interest | 28607567 | The outbreak fingolimod cardiovascular side effects in relapsing-remitting multiple sclerosis patient: A longitudinal study in an Iranian population |
| Οut of scope | 28639536 | Recurrent natalizumab-related aseptic meningitis in a patient with multiple sclerosis |
| No outcomes of interest | 28641055 | Long-term safety evaluation of natalizumab for the treatment of multiple sclerosis |
| Study type | 28749311 | Reconstitution of the peripheral immune repertoire following withdrawal of fingolimod |
| No outcomes of interest | 28751099 | Efficacy and Safety of Delayed-release Dimethyl Fumarate for Relapsing-remitting Multiple Sclerosis in Prior Interferon Users: An Integrated Analysis of DEFINE and CONFIRM |
| No outcomes of interest | 28770420 | Efficacy and Tolerability of Delayed-release Dimethyl Fumarate in Black, Hispanic, and Asian Patients with Relapsing-Remitting Multiple Sclerosis: Post Hoc Integrated Analysis of DEFINE and CONFIRM |
| No outcomes of interest | 28780745 | Effectiveness and Safety of Dimethyl Fumarate Treatment in Relapsing Multiple Sclerosis Patients: Real-World Evidence |
| Οut of scope | 28791286 | Autoimmune Thrombotic Thrombocytopenic Purpura: Two Rare Cases Associated with Juvenile Idiopathic Arthritis and Multiple Sclerosis |
| No outcomes of interest | 28835401 | Alemtuzumab CARE-MS I 5-year follow-up: Durable efficacy in the absence of continuous MS therapy |
| No outcomes of interest | 28835403 | Alemtuzumab CARE-MS II 5-year follow-up: Efficacy and safety findings |
| No outcomes of interest | 28861122 | The role of natalizumab in the treatment of multiple sclerosis: benefits and risks |
| No outcomes of interest | 28870107 | Safety and efficacy of cladribine tablets in patients with relapsing-remitting multiple sclerosis: Results from the randomized extension trial of the CLARITY study |
| No outcomes of interest | 28893804 | Immune thrombocytopenic purpura associated with fingolimod |
| No outcomes of interest | 28906152 | Relapse outcomes, safety, and treatment patterns in patients diagnosed with relapsing-remitting multiple sclerosis and initiated on subcutaneous interferon β-1a or dimethyl fumarate: a real-world study |
| No outcomes of interest | 28960496 | Synthesis and Functional Investigations of Computer Designed Novel Cladribine-Like Compounds for the Treatment of Multiple Sclerosis |
| No outcomes of interest | 28976408 | Real-World Safety and Patient Profile of Fingolimod in Relapsing-Remitting Multiple Sclerosis: A Prospective Analysis in Buenos Aires, Argentina |
| No outcomes of interest | 29057262 | Relationship between Interferon Beta-1A Administration and Intracranial Vascular Tone Regulation in Patients with Relapsing-Remitting Multiple Sclerosis: A Pilot Study |
| Study type | 29138536 | Spotlight on siponimod and its potential in the treatment of secondary progressive multiple sclerosis: the evidence to date |
| No outcomes of interest | 29212866 | Acquired haemophilia A complicating alemtuzumab therapy for multiple sclerosis |
| No outcomes of interest | 29256149 | Cardiovascular profile improvement during Natalizumab treatment |
| No outcomes of interest | 29359614 | Life-threatening autoimmune warm hemolytic anemia following treatment for multiple sclerosis with alemtuzumab |
| No outcomes of interest | 29359617 | Simultaneous early-onset severe autoimmune hemolytic anemia and albuminuria during alemtuzumab treatment for multiple sclerosis |
| No outcomes of interest | 29389745 | Safety and Efficacy of Rituximab: Experience of a Single Multiple Sclerosis Center |
| Οut of scope | 29397790 | Potential neuroprotective effect of Fingolimod in multiple sclerosis and its association with clinical variables |
| Οut of scope | 29399047 | Decreased platelet number in multiple sclerosis during alemtuzumab infusion: a common, transient and clinically silent phenomenon |
| Study type | 29484976 | New Life to an Old Treatment: Pegylated Interferon Beta 1a in the Management of Multiple Sclerosis |
| No outcomes of interest | 29526118 | First-line disease-modifying drugs in relapsing-remitting multiple sclerosis: an Italian real-life multicenter study on persistence |
| No outcomes of interest | 29576505 | Siponimod versus placebo in secondary progressive multiple sclerosis (EXPAND): a double-blind, randomised, phase 3 study |
| Οut of scope | 29617694 | Soluble Receptor for Advanced Glycation End Products (sRAGE) is Up-Regulated in Multiple Sclerosis Patients Treated with Interferon β-1a |
| Οut of scope | 29656444 | Predicting therapeutic response to fingolimod treatment in multiple sclerosis patients |
| No outcomes of interest | 29664454 | Clinical effectiveness and safety of fingolimod in relapsing remitting multiple sclerosis in Western Iran |
| Νot population of interest | 29681490 | Safety and Efficacy of Delayed-Release Dimethyl Fumarate in Pediatric Patients With Relapsing Multiple Sclerosis (FOCUS) |
| No outcomes of interest | 29695594 | Subcutaneous ofatumumab in patients with relapsing-remitting multiple sclerosis: The MIRROR study |
| No outcomes of interest | 29696498 | Alemtuzumab as rescue therapy in a cohort of 50 relapsing-remitting MS patients with breakthrough disease on fingolimod: a multi-center observational study |
| Study type | 29719400 | New horizons for multiple sclerosis therapeutics: milestones in the development of ocrelizumab |
| Οut of scope | 29730587 | Neutropenia with fatal outcome in a multiple sclerosis patient 23 days after alemtuzumab infusion |
| No outcomes of interest | 29758075 | Effectiveness and safety of Rituximab in multiple sclerosis: an observational study from Southern Switzerland |
| No outcomes of interest | 29844796 | Phase IV study of retention on fingolimod versus injectable multiple sclerosis therapies: a randomized clinical trial |
| No outcomes of interest | 29897610 | Ocrelizumab: its efficacy and safety in multiple sclerosis |
| No outcomes of interest | 29906666 | Real-life persistence and tolerability with dimethyl fumarate |
| No outcomes of interest | 29911471 | Successful implementation of an automated electronic support system for patient safety monitoring: The alemtuzumab in multiple sclerosis safety systems (AMS3) study |
| No outcomes of interest | 29927805 | Safety and Effectiveness of Fingolimod in Real-World Multiple Sclerosis Portuguese Patients |
| No outcomes of interest | 29948245 | Two-year real-life efficacy, tolerability and safety of dimethyl fumarate in an Italian multicentre study |
| No outcomes of interest | 29968546 | The Matrix Metalloproteinases Panel in Multiple Sclerosis Patients Treated with Natalizumab: A Possible Answer to Natalizumab Non- Responders |
| Study type | 29970109 | Teriflunomide attenuates neuroinflammation-related neural damage in mice carrying human PLP1 mutations |
| No outcomes of interest | 30022464 | Safety and Efficacy of Dimethyl Fumarate in Multiple Sclerosis: An Italian, Multicenter, Real-World Study |
| No outcomes of interest | 30036854 | Treatment retention on fingolimod compared with injectable multiple sclerosis therapies in African-American patients: A subgroup analysis of a randomized phase 4 study |
| Study type | 30050372 | Spotlight on daclizumab: its potential in the treatment of multiple sclerosis |
| No outcomes of interest | 30181778 | Long-term outcomes of peginterferon beta-1a in multiple sclerosis: results from the ADVANCE extension study, ATTAIN |
| No outcomes of interest | 30206820 | 72-Week Safety and Tolerability of Dimethyl Fumarate in Japanese Patients with Relapsing-remitting Multiple Sclerosis: Analysis of the Randomised, Double Blind, Placebo-Controlled, Phase III APEX Study and its Open-Label Extension |
| Νot population of interest | 30207920 | Trial of Fingolimod versus Interferon Beta-1a in Pediatric Multiple Sclerosis |
| No outcomes of interest | 30320947 | Dimethyl fumarate and teriflunomide for multiple sclerosis in a real-life setting: a French retrospective cohort study |
| Οut of scope | 30337289 | Development of acquired haemophilia A in a patient treated with alemtuzumab for multiple sclerosis |
| No outcomes of interest | 30348586 | Efficacy and safety of teriflunomide in Asian patients with relapsing forms of multiple sclerosis: A subgroup analysis of the phase 3 TOWER study |
| Οut of scope | 30354753 | Multiple Sclerosis Drug Fingolimod Induces Thrombotic Microangiopathy in Deoxycorticosterone Acetate/Salt Hypertension |
| Οut of scope | 30369266 | Neutrophil-released enzymes can influence composition of circulating immune complexes in multiple sclerosis |
| No outcomes of interest | 30419510 | Treating the ineligible: Disease modification in people with multiple sclerosis beyond NHS England commissioning policies |
| No outcomes of interest | 30446966 | Efficacy and safety of alemtuzumab versus fingolimod in RRMS after natalizumab cessation |
| No outcomes of interest | 30511679 | Efficacy and Safety of Teriflunomide in Chinese Patients with Relapsing Forms of Multiple Sclerosis: A Subgroup Analysis of the Phase 3 TOWER Study |
| No outcomes of interest | 30539030 | Safety and Efficacy of Rituximab in Multiple Sclerosis: A Retrospective Observational Study |
| No outcomes of interest | 30616596 | A randomized placebo-controlled trial of delayed-release dimethyl fumarate in patients with relapsing-remitting multiple sclerosis from East Asia and other countries |
| Νot population of interest | 30635477 | Rituximab vs placebo induction prior to glatiramer acetate monotherapy in multiple sclerosis |
| No outcomes of interest | 30702749 | The first reported case of drug-induced hemolytic anemia caused by dimethyl fumarate in a patient with multiple sclerosis |
| No outcomes of interest | 30755869 | Glatiramer Acetate-associated Refractory Immune Thrombocytopenic Purpura |
| Οut of scope | 30785074 | Potential mechanisms of action related to the efficacy and safety of cladribine |
| No outcomes of interest | 30785358 | Immune thrombocytopenia in alemtuzumab-treated MS patients: Incidence, detection, and management |
| Study type | 30801313 | New Warning for the Multiple Sclerosis Drug Alemtuzumab |
| No outcomes of interest | 30844611 | Ocrelizumab infusion experience in patients with relapsing and primary progressive multiple sclerosis: Results from the phase 3 randomized OPERA I, OPERA II, and ORATORIO studies |
| No outcomes of interest | 30849681 | Efficacy and safety of alemtuzumab in Korean multiple sclerosis patients |
| No outcomes of interest | 30863891 | Efficacy and safety of alemtuzumab in a real-life cohort of patients with multiple sclerosis |
| No outcomes of interest | 30885374 | Safety of cladribine tablets in the treatment of patients with multiple sclerosis: An integrated analysis |
| No outcomes of interest | 30944584 | Real-life outcomes of teriflunomide treatment in patients with relapsing multiple sclerosis: TAURUS-MS observational study |
| Study type | 31005729 | Teriflunomide real-world evidence: Global differences in the phase 4 Teri-PRO study |
| Study type | 31144287 | Siponimod: First Global Approval |
| Study type | 31191177 | Stabilization Without Rituximab After Disease Activation in an Alemtuzumab-Treated Patient with Multiple Sclerosis and a Literature Overview |
| No outcomes of interest | 31213818 | Drug-use patterns and severe adverse events with disease-modifying drugs in patients with multiple sclerosis: a cohort study based on German claims data |
| No outcomes of interest | 31218077 | Efficacy and safety of dimethyl fumarate in treatment-naïve Japanese patients with multiple sclerosis: Interim analysis of the randomized placebo-controlled study |
| No outcomes of interest | 31252364 | Acute myocardial infarction associated with initial alemtuzumab infusion cycle in relapsing-remitting multiple sclerosis |
| Οut of scope | 31276502 | RebiQoL: A randomized trial of telemedicine patient support program for health-related quality of life and adherence in people with MS treated with Rebif |
| No outcomes of interest | 31285147 | Safety and efficacy of opicinumab in patients with relapsing multiple sclerosis (SYNERGY): a randomised, placebo-controlled, phase 2 trial |
| No outcomes of interest | 31299922 | Use of glatiramer acetate between 2010-2015: effectiveness, safety and reasons to start GA as first or second line treatment in Swiss multiple sclerosis patients |
| No outcomes of interest | 31353999 | A retrospective observational study of rituximab treatment in multiple sclerosis patients in Cyprus |
| Νot population of interest | 31462595 | Treatment with Rituximab in the Acute Phase of Relapsing Remitting Multiple Sclerosis |
| No outcomes of interest | 31482275 | The Putative Association of TOB1-AS1 Long Non-coding RNA with Immune Tolerance: A Study on Multiple Sclerosis Patients |
| No outcomes of interest | 31492651 | Safety and efficacy of ozanimod versus interferon beta-1a in relapsing multiple sclerosis (SUNBEAM): a multicentre, randomised, minimum 12-month, phase 3 trial |
| No outcomes of interest | 31492652 | Safety and efficacy of ozanimod versus interferon beta-1a in relapsing multiple sclerosis (RADIANCE): a multicentre, randomised, 24-month, phase 3 trial |
| No outcomes of interest | 31551258 | The natalizumab wearing-off effect: End of natalizumab cycle, recurrence of MS symptoms |
| No outcomes of interest | 31555463 | Alemtuzumab following natalizumab in highly active paediatric-onset multiple sclerosis |
| Οut of scope | 31558140 | Siponimod (BAF-312) Attenuates Perihemorrhagic Edema And Improves Survival in Experimental Intracerebral Hemorrhage |
| Οut of scope | 31562558 | No evidence of disease activity including cognition (NEDA-3 plus) in naïve pediatric multiple sclerosis patients treated with natalizumab |
| No outcomes of interest | 31603362 | Siponimod in the treatment of multiple sclerosis |
| No outcomes of interest | 31631980 | Clinical evaluation of dimethyl fumarate for the treatment of relapsing-remitting multiple sclerosis: efficacy, safety, patient experience and adherence |
| No outcomes of interest | 31650471 | Treatment with Dimethyl Fumarate Enhances Cholinergic Transmission in Multiple Sclerosis |
| No outcomes of interest | 31654272 | Efficacy and Safety of Alemtuzumab in Patients of African Descent with Relapsing-Remitting Multiple Sclerosis: 8-Year Follow-up of CARE-MS I and II (TOPAZ Study) |
| No outcomes of interest | 31729968 | Analysis of cardiac monitoring and safety data in patients initiating fingolimod treatment in the home or in clinic |
| No outcomes of interest | 31733426 | Alemtuzumab treatment of multiple sclerosis in real-world clinical practice: A report from a single Italian center |
| No outcomes of interest | 31843437 | Short term real-world Fingolimod efficacy and safety in Emirati patients with multiple sclerosis |
| No outcomes of interest | 31901758 | The 5-year Tysabri global observational program in safety (TYGRIS) study confirms the long-term safety profile of natalizumab treatment in multiple sclerosis |
| No outcomes of interest | 31903032 | A 12-month, Open Label, Multicenter Pilot Study Evaluating Fingolimod Treatment in terms of Patient Satisfaction in Relapsing Remitting Multiple Sclerosis Patients - FINE Trial |
| No outcomes of interest | 32117551 | Effectiveness and side effects of dimethyl fumarate in multiple sclerosis after 12 months of follow up: An Iranian clinical trial |
| No outcomes of interest | 32145001 | Alemtuzumab for relapsing multiple sclerosis in clinical practice: A four-year retrospective one-center study |
| No outcomes of interest | 32181303 | Data of safety in a single-center alemtuzumab treated population |
| No outcomes of interest | 32241823 | Autoimmune hemolytic anemia, demyelinating relapse, and AQP1 antibodies after alemtuzumab infusion |
| Οut of scope | 33324905 | Signatures of immune reprogramming in anti-CD52 therapy of MS: markers for risk stratification and treatment response |
